# Sinapis Semen: A review on phytochemistry, pharmacology, toxicity, analytical methods and pharmacokinetics

**DOI:** 10.3389/fphar.2023.1113583

**Published:** 2023-04-12

**Authors:** Rui Dang, Huida Guan, Changhong Wang

**Affiliations:** The MOE Key Laboratory for Standardization of Chinese Medicines, Shanghai R&D Centre for Standardization of Chinese Medicines, Institute of Chinese Materia Medica, Shanghai University of Traditional Chinese Medicine, Shanghai, China

**Keywords:** Sinapis Semen, phytochemistry, pharmacology, toxicity, analytical methods, pharmacokinetics

## Abstract

Sinapis Semen (SS), the dried mature seed of *Sinapis alba* L. and *Brassica juncea* (L.) Czern. et Coss., is one of the traditional Chinese medicinal materials with a wide range of pharmacological effects being used for asthma, cough and many other ailments. SS is also widely used in food agriculture, medicine and other industries in North America and South Asia. More recently, the research on SS has gradually intensified and increased. However, there is no systematic review of SS. In this review, through literature exploration and analysis, the research advance on phytochemistry, pharmacology, toxicity, analytical methods and pharmacokinetics of SS was aggregated initially. Total 144 compounds have been isolated and identified from SS. Among them, glucosinolates and their hydrolysates and volatile oils are the main active ingredients and important chemical classification markers. SS has a wide range of pharmacological effects, especially in cough suppressing, asthma calming, anti-inflammatory, neuroprotective, cardiovascular protective, inhibiting androgenic effects, anti-tumor, and skin permeation promoting effects. Sinapine and sinapic acid are the main active ingredients of SS for its medicinal effects. However, SS has a strong skin irritation, presumably related to the time of application, the method of processing, and original medicinal plants. This review will provide useful data for the follow-up research and safe and reasonable clinical application of SS.

## Graphical Abstract

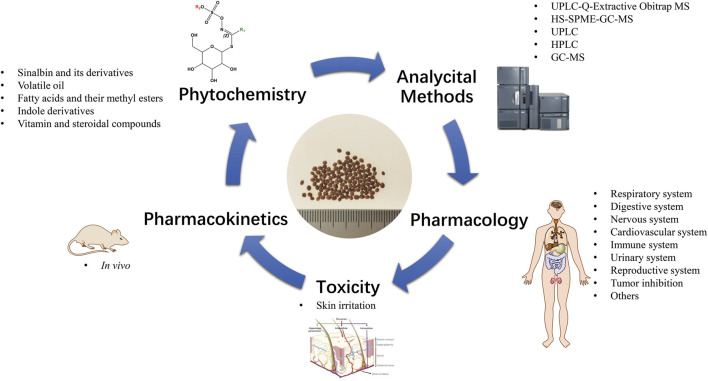

## 1 Introduction

Sinapis Semen (SS), the dried mature seed of *Sinapis alba* L. and *Brassica juncea* (L.) Czern. et Coss., is widely used in China, North America and South Asia in food agriculture, medicine and other industries (Nicácio et al., 2021). SS has the function of warming the lung and resolving phlegm, promoting qi and removing stasis, dredging collaterals and alleviating pain (Wu et al., 2022). SS has a long history of medicinal use, functioning as treating cough with cold phlegm, chest distension and pain, phlegm stagnation in the meridians and collaterals, numbness and pain in the joints, phlegm-dampness flow, gangrene and swelling pain. SS was first published as name of “Jie” in the *Miscellaneous Records of Famous Physicians* (Sun, 2015). The processing method of SS is mostly stir-frying to yellow (Liang et al., 2007; Yang et al., 2010).

SS is often combined with other herbs in clinic for treatment of bronchial asthma and other respiratory diseases (Liu et al., 2018). The treatment of winter diseases in summer is a characteristic therapeutic strategy of traditional Chinese medicine. It refers to the treatment method of giving targeted treatment in summer to improve the body’s ability to resist diseases, thus to reduce or eliminate the diseases that tend to occur or aggravate in winter. The most common treatment method for the winter disease cured in summer is herbal acupuncture point paste. SS is used as a basic medicine for the winter disease cured in summer due to its skin penetration enhancing effect (Fang et al., 2010; Ruan et al., 2019).

Up to now, more than 140 chemical components of SS have been identified. Glucosinolates and their hydrolysates are the main components of SS that exert medicinal effects (Wang et al., 2015). Modern pharmacological studies have shown that SS has anti-proliferative, pro-apoptotic, antioxidant, anti-cancer, anti-tumor and anti-bacterial effects, etc. (Boscaro et al., 2018). The glucosinolates in SS and their degradation products have anti-cough and asthma, liver protection, antioxidant, anti-cancer and other effects (Zhang et al., 2015). SS has been used for asthma, cough, hypertension, hyperglycemia, hyperlipidemia, testicular damage, prostate enlargement, fatty liver, tumors, and herniated lumbar disc (Ou, 2002; Zhou et al., 2014). Many studies have been focused on the biological applications of SS. Myrosinase is the only enzyme that catalyze the cleavage of the S-glycosidic bond using ascorbate as a cofactor and a reaction mechanism that retains the anomeric configuration at the cleavage. Glucosinolates are hydrolyzed by myrosinase, and depending on the structure of side chain, the presence of additional proteins and cofactors generates components such as isothiocyanates and nitriles. The products obtained from hydrolysis exert a variety of pharmacological effects (Halkier and Gershenzon, 2006; Bhat and Vyas, 2019). A small number of scholars have studied the pharmacokinetics of SS. In addition, SS is strongly irritative to skin when it is for external use, manifested as local pain, red skin, itching, sting, blisters and infection (Wu et al., 2016). But there is no systematic review of the phytochemistry, analytical methods, pharmacology, toxicity and pharmacokinetics of SS. This paper reviews the phytochemistry, pharmacological effects, toxicity and *in vivo* metabolic processes of SS from the perspective of ethnopharmacology in published articles in recent decades to provide assistance in the development of SS safety and further research.

## 2 Ethnopharmacological use

With a long history of medicinal use for more than 2,000 years in China, SS is widely used for the treatment of cough and asthma caused by phlegm retention, fullness and pain in chest and hypochondrium, nausea and vomiting, aphasia from apoplexy, limb paralysis and numbness, barbiers, dorsal furuncle, swelling and pain from bruises (Yu, 2005).

SS can be taken orally, and is also for external use. Since ancient times, with the application of SS more and more widely, more processing methods and effectiveness have been gradually discovered. In *Lei Gong Pao Zhi Yao Xing Jie*, it was written that, “It can treat parasitic tympanties when being processed with vinegar.” According to *Ben Cao Zheng Yao*, taking the liquor with SS, nausea will disappear, and applying SS with vinegar, carbuncle toxin can be treated. In addition to being stir-fried, SS can also be processed with liquor or vinegar.

In clinical practice, SS is usually applied with other herbs. It can be combined with Raphani Semen to treat cough, and with Myrrha to treat arthralgia. When it is used with *Lycopodii herba*, bone ache can be relieved, and with *Chuanxiong rhizoma*, headache can be eased (Liu and Shi, 2017). The classical formulations containing SS handed down from ancient medical books or ethnic medical experience are now widely used in clinic.

San Zi Yang Qin Decoction (Duan, 2007) is a traditional famous prescription to relieve cough, prevent asthma, and eliminate phlegm. Ma Xin Gan Shi Decoction is a proved prescription to treat asthma, it is beneficial to diffuse lung qi, tonify spleen, reduce phlegm and fluid retention, and relieve cough and asthma (Zhou et al., 2015). Bai Jie Zi Powder, composed of SS, Corydalis Rhizoma, Asari Radix et Rhizoma, and Kansui Radix, can be used to treat bronchial asthma by relieving cough and asthma, enhancing the body resistance and tonifying qi effectively (Liu et al., 2018). Due to the multiple applications, now SS has been made into paste, patch, decoction, powder, pill and medicinal liquor.

## 3 Phytochemistry

After years of phytochemical research, 144 compounds have been isolated and identified from SS. The compounds (**1**–**144**) of SS reported in the literatures were listed in (Supplementary Table S1). SS is rich in glucosinolates and their hydrolysates, which are the main components with pharmacological activity. Sinapine (**1**) and 4-hydroxybenzoylcholine (**21**) often used as an indicator component for the authentification of SS. Sinigrin (**4**) and sinalbin (**5**) are used as quality control components in SS. The extract of SS is soluble in water or alcohol. Glucosinolates and their hydrolysates are water-soluble components, and the volatile oil, fatty acids and their methyl esters are fat-soluble components (Buskov et al., 2000). Glucosinolates in SS are easy to be degraded under specific conditions by myrosase (mustard enzymes). The processing method of frying kills enzymes and protects glycosides (Zhang et al., 2015). Both sinapine thiocyanate (**3**) and 4-hydroxybenzyl cyanide (**18**) increased after frying (Liang et al., 2007; Yang et al., 2010). The volatile components (compound **24**–**86**) of SS are mainly isothiocyanates, nitriles, alkanes, olefins, terpenoids, etc. (Cai et al., 2014). Erucic acid (**97**), methyl erucate (**116**), linoleic acid (**90**) and methyl linoleate (**111**) are the components of fatty acids and fatty acid methyl esters with high content (Zhang and Wang, 2006). There are differences in the composition of *S. alba* and *B. juncea*, and the response value of sinapine thiocyanate (**3**) in *S. alba* is higher than that of *B. juncea*. Sinigrin (**4**) and sinalbin (**5**) are thioglucosides specific to *S. alba* and *B. juncea*, respectively (Huang et al., 2020). Sinalbin (**5**) and 4-hydroxybenzoylcholine (**21**) are unique components of *S. alba* (Zhang et al., 2010).

### 3.1 Glucosinolates and their hydrolysates

**FIGURE 1:**
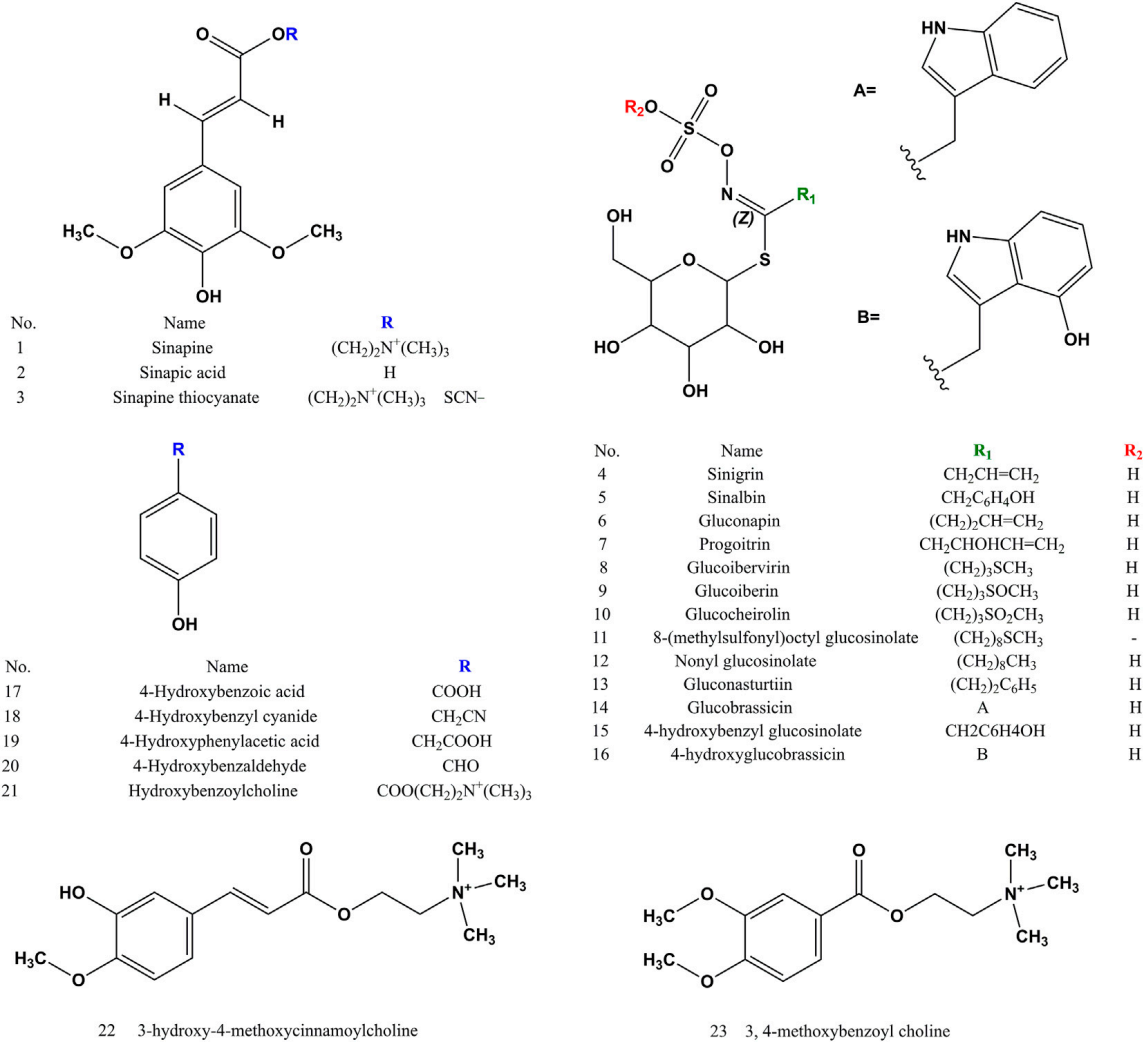
Chemical structures of glucosinolates and their hydrolysates identified from SS (compound **1**–**23**).

The basic skeleton of the compounds (**1**–**3**) contained acrylate and 2,6-dimethoxyphenol at C-3. Sinapine (**1**) was formed by the cleavage of one ethyl trimethylammonium under enzymatic, alkaline and high-temperature conditions to sinapic acid (**2**), and the addition of one thiocyanate ion to sinapine thiocyanate (**3**). The compounds (**4**–**16**) are thioglucose analogs. Glucosinolates are characteristic of cruciferous seeds, and such components are composed of a sugar-containing group, a sulfate group, and a variable non-sugar side chain (R_1_). Sinalbin (**5**) is the most abundant thioglucoside component in SS. Sinapine (**1**), sinapic acid (**2**), sinapine thiocyanate (**3**) are hydrolysates of sinalbin (**5**). The compounds (**17**–**21**) are degradation products of sinalbin (**5**). 3-Hydroxy-4-methoxycinnamoylcholine (**22**) was produced by methylation, demethylation, and demethoxylation of sinapine (**1**). 3,4-Methoxybenzoyl choline (**23**) was generated by the methylation and methoxylation of 4-hydroxybenzoylcholine (**21**) (Zhang et al., 2015). The main chemical structures of glucosinolates and their hydrolysates are shown in Figure 1.

### 3.2 Volatile oils

**FIGURE 2:**
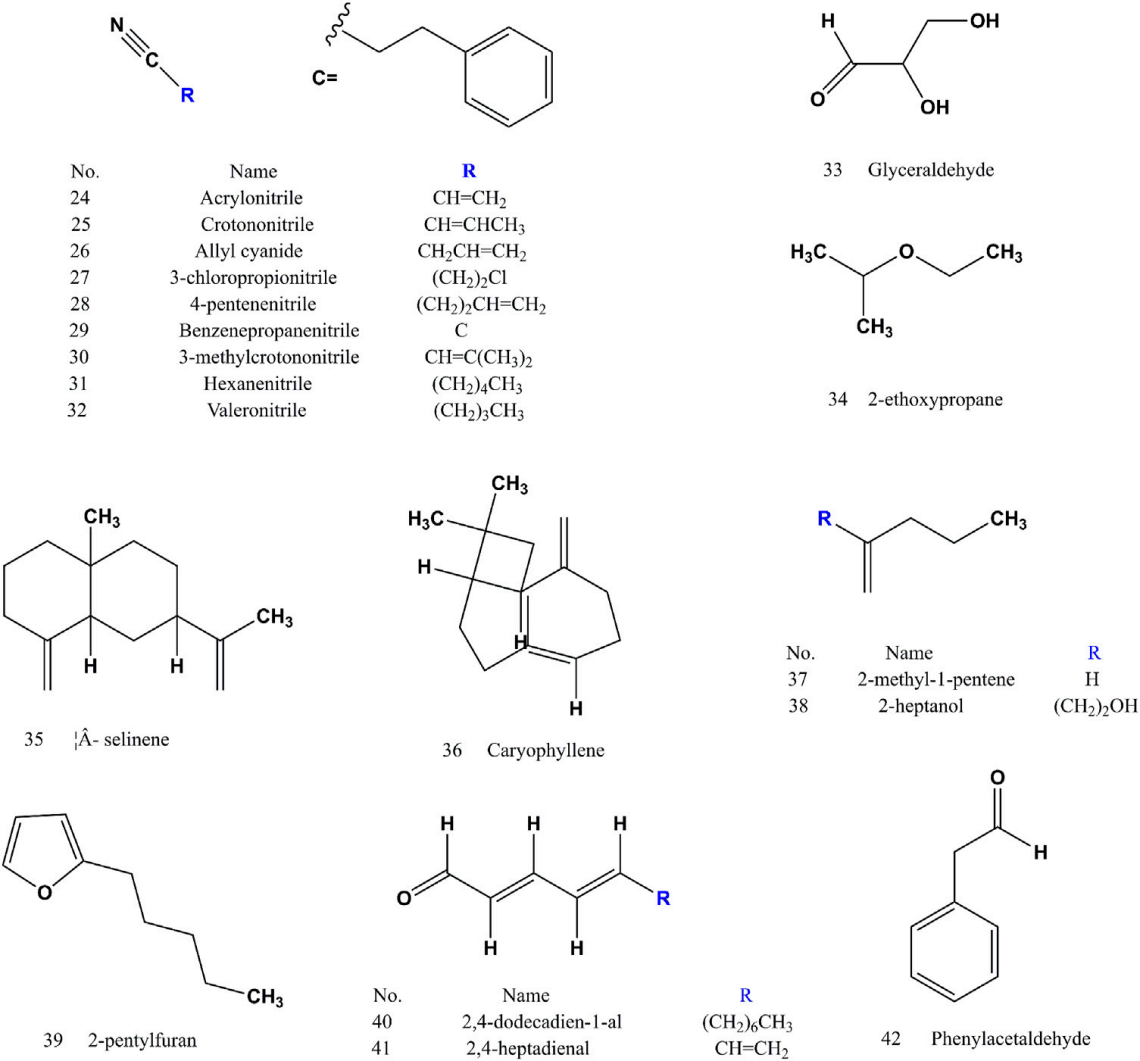
Partial chemical structures of volatile oils identified from SS (compound **24**–**42**).

**FIGURE 3:**
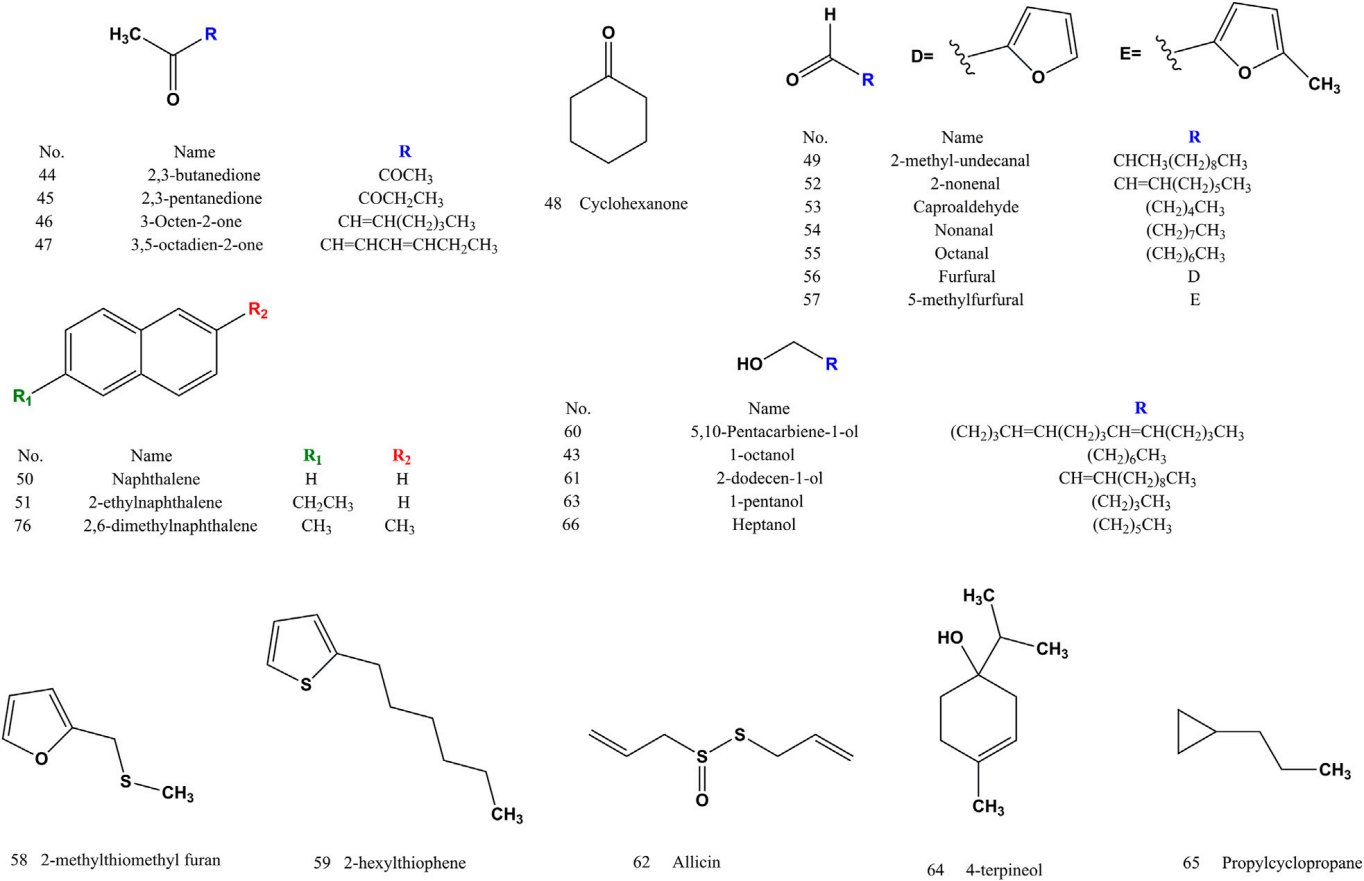
Partial chemical structures of volatile oils identified from SS (compound **43**–**66**).

**FIGURE 4:**
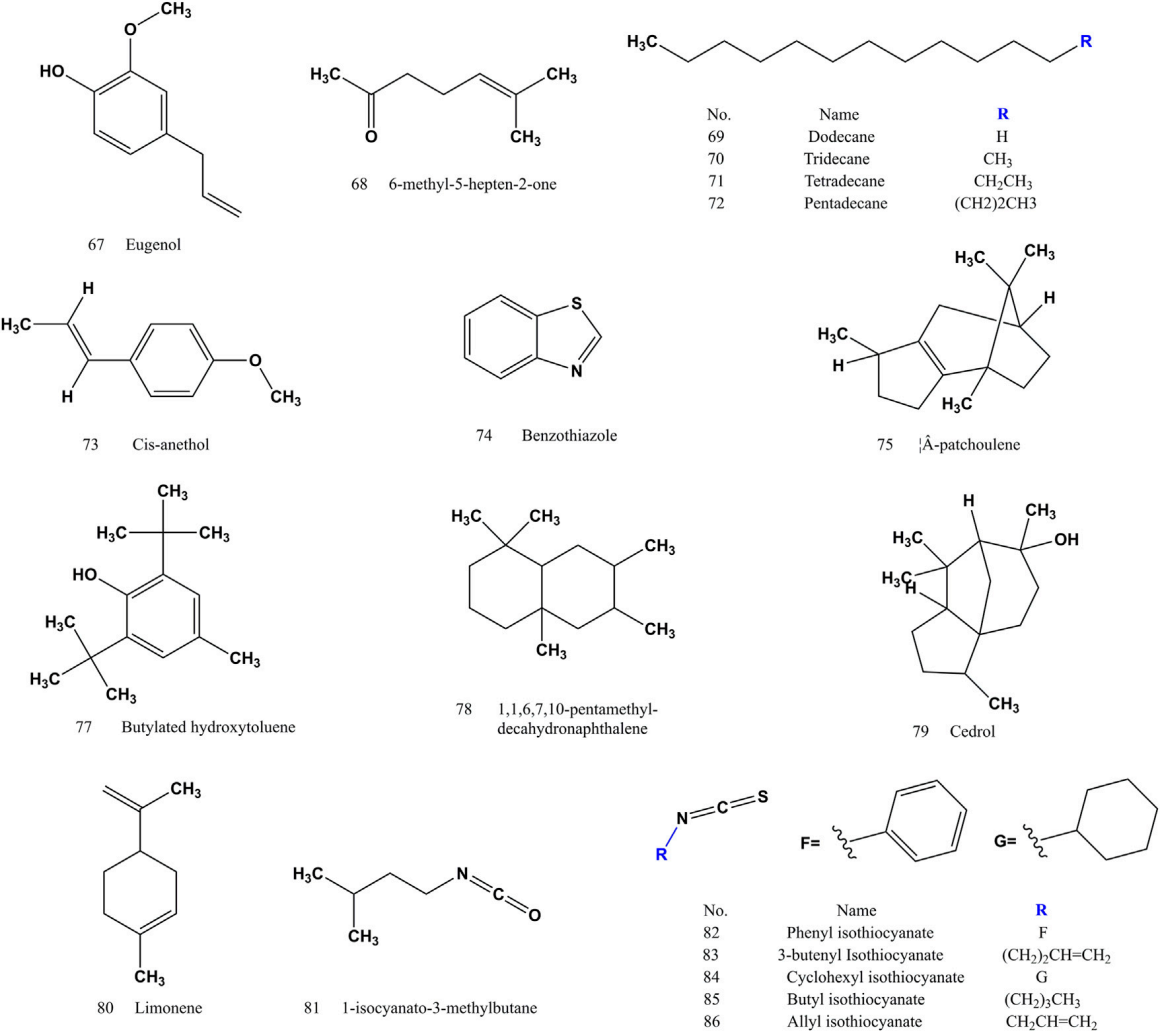
Partial chemical structures of volatile oils identified from SS (compound **67**–**86**).

Volatile oils (compound **24**–**86**) (Figures 2–4) are the active ingredient of SS to relieve cough and asthma and expectorant. The nitriles and isothiocyanates in the volatile oil are derived from the degradation of glucosinolates by the action of mustard enzymes. Isothiocyanates (compound **82**–**86**) are a naturally occurring class of compounds in the cruciferous family that all contain thiocyanogenic group. Allyl isothiocyanate (**86**) accounted for 89% of the total volatile oil (Wu et al., 2010).

### 3.3 Fatty acids and their methyl esters

**FIGURE 5:**
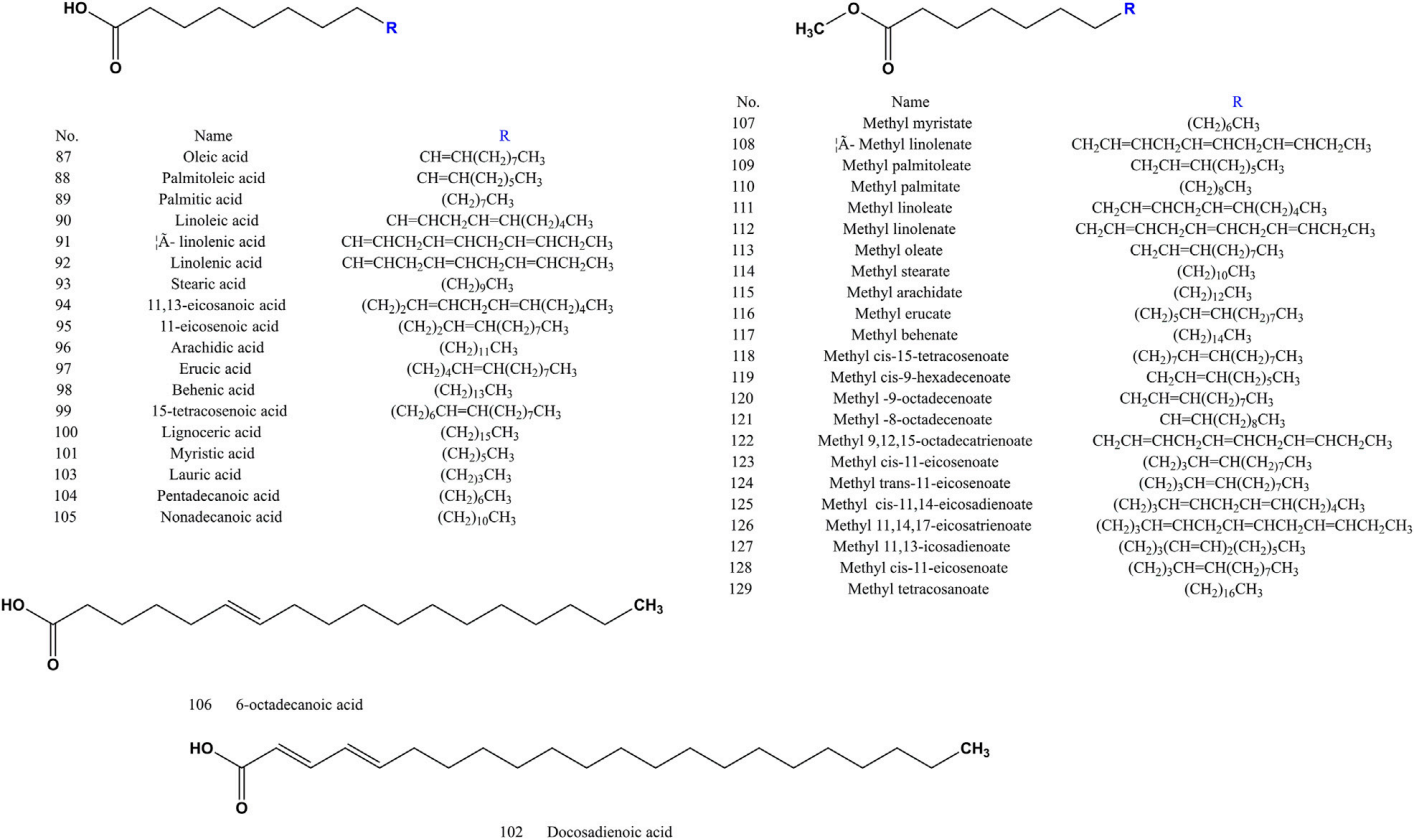
Chemical structures of fatty acids and their methyl esters identified from SS (compound **87**–**129**).

Most researchers have used supercritical CO_2_ extraction and GC-MS to extract and analyze the fatty acids and fatty acid methyl esters in SS. The chemical structures of fatty acids (compound **87**–**106**) and their methyl esters (compound **107**–**129**) in SS are shown in Figure 5. Fatty acid methyl ester is produced by methylation of fatty acid, and fatty acid methyl ester is an important chemical intermediate.

### 3.4 Indole derivatives

**FIGURE 6:**
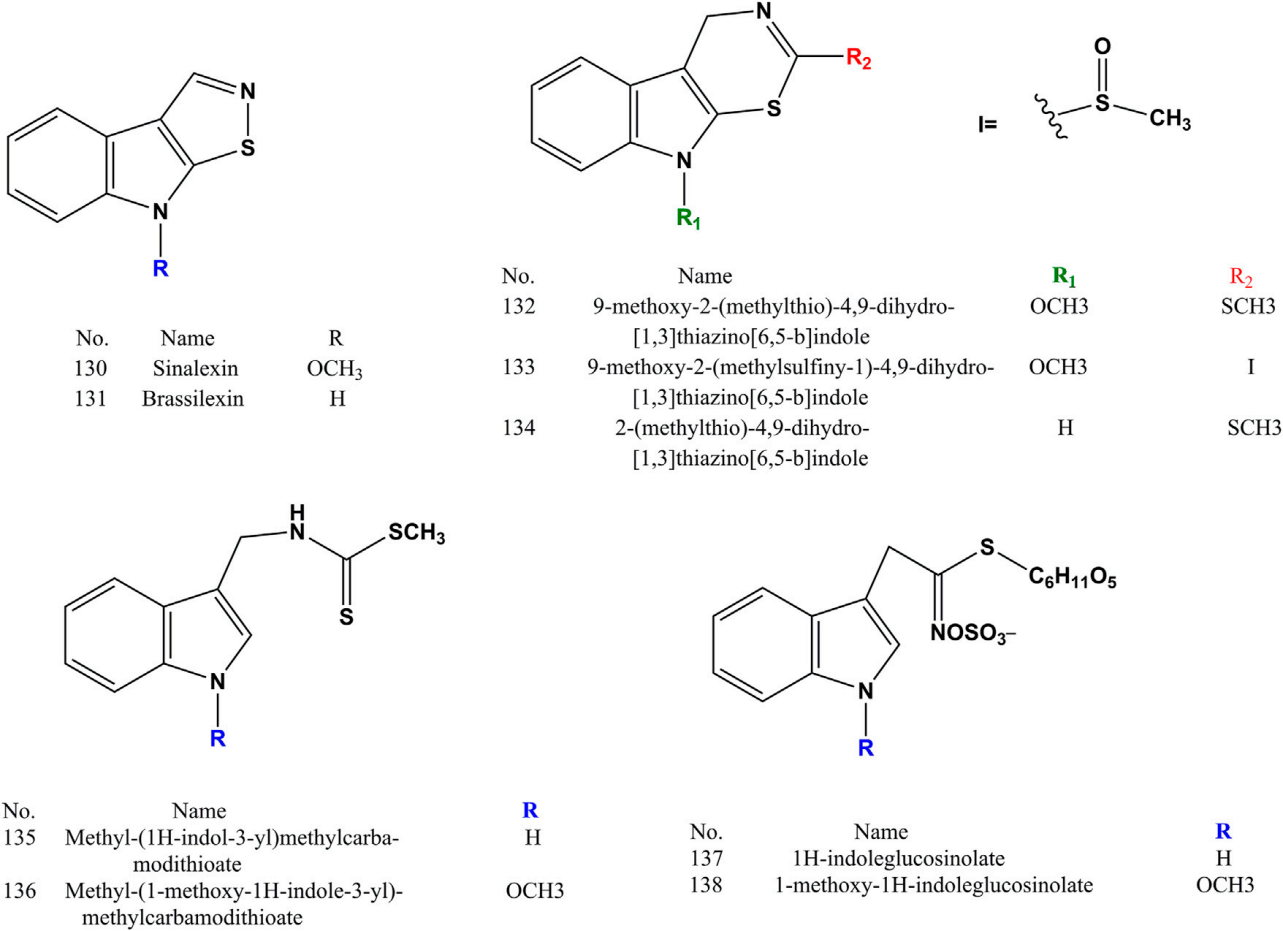
Chemical structures of indole derivatives identified from SS (compound **130**–**138**).

Nine indole derivatives (compound **130**–**138**) contained in SS are currently reported in the literature. Indole derivatives are aromatic heterocyclic compounds whose chemical structures are formed by the juxtaposition of a benzene ring with a pyrrole ring, as shown in Figure 6. At present, the pharmacological effects of indole derivatives in SS have not been reported much.

### 3.5 Others

**FIGURE 7:**
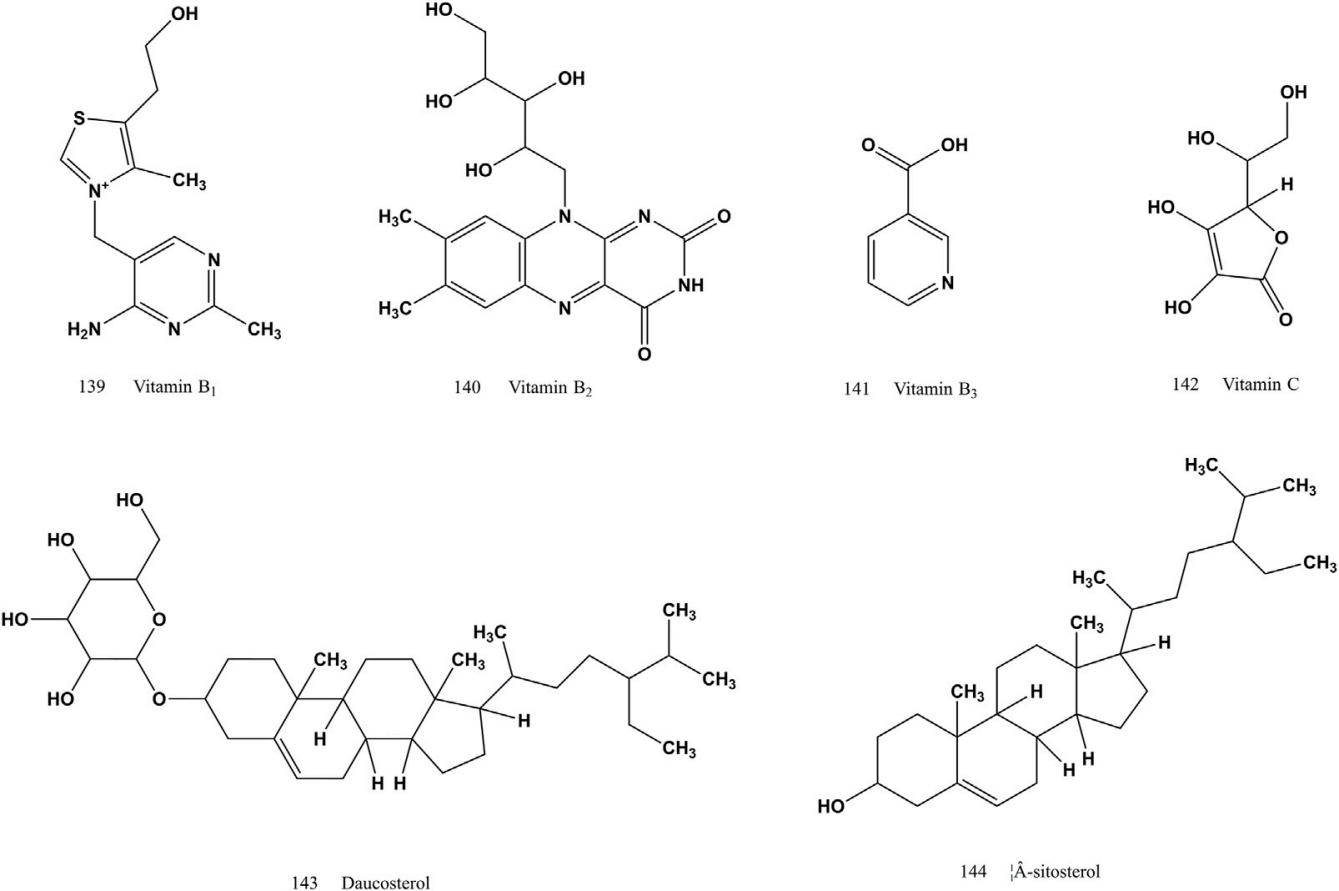
Chemical structures of vitamin and steroidal compounds identified from SS (compound **139**–**144**).

Vitamin B_1_ (**139**), vitamin B_2_ (**140**), vitamin B_3_ (**141**), vitamin C (**142**), daucosterol (**143**) and β-sitosterol (**144**) are the vitamin and steroidal compounds reported in the literature so far in SS (Figure 7). The vitamin B family is mainly able to maintain the normal function of the nervous system and immune system, and vitamin C (**142**) mainly has antioxidant and anti-scorbutic effects. Daucosterol (**143**) can prevent dry skin and promote growth and development. β-Sitosterol (**144**) can lower serum cholesterol.

Comprehensively, glucosinolates and their hydrolysates are particular components in SS. Depending on structure, glucosinolates are hydrolyzed by myrosinase into different products. Although the intact glucosinolate is not prominent in pharmacological activity, its hydrolysis products exhibit diverse biological activities. Glucosinolates are of great importance for both agriculture and Chinese medicine. Therefore, glucosinolates is a kind of component in SS worthy of future in-depth research, especially in its occurrence and transformation mechanism.

## 4 Pharmacology

**FIGURE 8:**
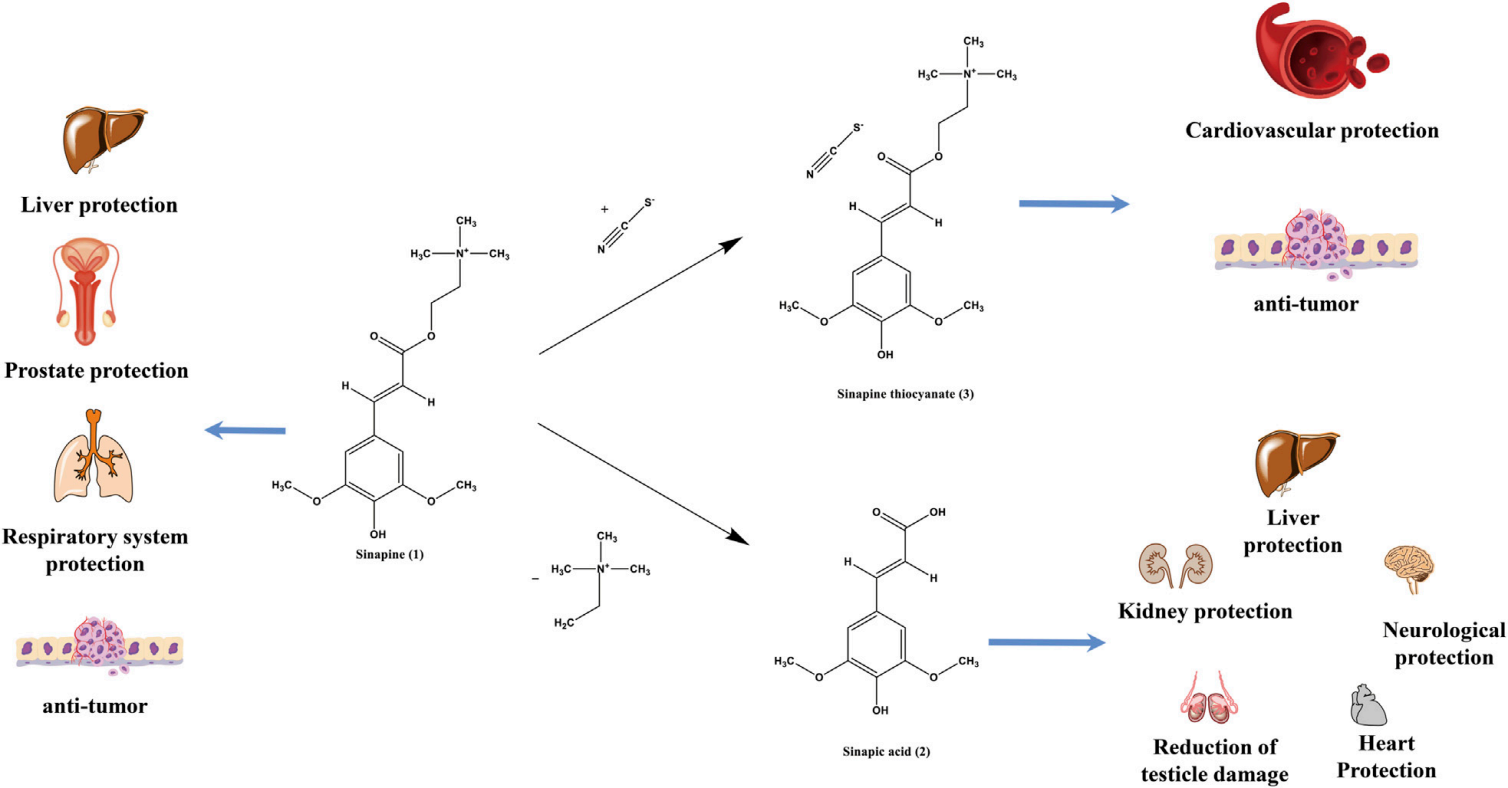
Pharmacological effects of Sinapine (**1**), sinapic acid (**2**) and sinapine thiocyanate (**3**).

In recent decades, the various traditional uses of SS have attracted extensive attention from scientists and have been studied for their wide range of physiological and pharmacological properties. SS extracts or active ingredients have demonstrated various pharmacological activities such as anti-asthmatic, anti-cough, liver protection, weight reduction, heart protection, anti-androgenic effects, anti-inflammatory, anti-cancer, anti-tumor and antioxidant (Table 1), which have great potential for research and drug development in respiratory diseases, digestive diseases, neurological diseases, cardiovascular system, immune system diseases, tumors, and inflammatory diseases (Figure 8).

**TABLE 1:** Pharmacological effects of SS.

System	Effect	Mechanism	Extracts or compounds	Models	*In vitro/vivo*	Treatment	References
Respiratory	Airway allergy reduction	Reduce TGF-β expression, MAPK phosphorylation, MMP9 and MMP2 protease activity	SS patch	Guinea pig asthma model	*In vivo*	0.0625–0.5 mg ml^−1^	Li (2017)
		Downregulation of TGF-β1/Smad 3 pathway	SS patch	BALB/c Chronic asthma mice model	*In vivo*	0.5 g	Liu et al. (2017)
		Relaxes airway smooth muscle, increases lung and airway volume	Sinapine	Guinea pig asthma model	*In vivo*	7.4–74 mg kg^−1^	Wang et al. (2011)
	Cough suppressant inhibition	Inhibition of the receptors in the cough reflex arc or the vagus nerve of the afferent cough impulses	4-Hydroxybenzyl cyanide	Cough model	*In vivo*	0.031 g kg^−1^	Yu (2005)
Digestive	Liver protection	Inhibition of BRD4 expression	Sinapic acid	C57BL/6 mice, AML-12 cells	*In vivo, In vitro*	20 mg kg^−1^, 20 μM	Chu et al. (2021)
		Inhibition of liver steatosis	Sinapine	C57BL/6 mice	*In vivo*	500 mg kg^−1^	Li et al. (2019)
		NF-κB inhibits Nrf2/HO-1-mediated activation of antioxidant enzymes and apoptosis inhibition	Sinapic acid	Hepatotoxicity model	*In vivo*	20, 40 mg kg^−1^	Ahmad et al. (2021)
		Reduced expression of TGF-β1, Smad4, p-Smad 2/3/Smad 2/3, p-NF-κB-p65/NF-κB-p65, IL-1β, IL-6 and p-AKT/AKT	SS extract	Hepatic fibrosis model	*In vivo*	0.5, 1.0, and 2.0 g kg^−1^	Cao et al. (2018)
	Anti- adipocyte browning	Stimulation of mitochondrial biogenesis through AMPK, p38 MAPK and CREB pathways leads to white adipocyte browning	Sinapic acid	3T3-L1 cells	*In vitro*	1–20 μM	Bae and Kim (2020)
Nervous	Protective neurological activity	Activation of BDNF/TrkB/ERK signaling pathway	Sinapic acid	PC12 cells	*In vitro*	50–100 μM	Xue et al. (2021)
		Enhance cell viability and inhibit oxidative stress and endoplasmic reticulum stress	Sinapic acid	SH-SY5Y cells	*In vitro*	50–800 μM	Tungalag and Yang (2021)
		Promotes CREB mRNA transcription	Sinapic acid	PC12 cells	*In vitro*	100 μM	Xue et al. (2022)
Cardiovascular	Anti-hypertension	Inhibition of TNF-α production	Sinapine thiocyanate	Insulin resistance model	*In vivo*	10, 30, and 90 mg kg^−1^ d^−1^	Huang et al. (2018)
		Inhibition of NLRP3 inflammatory vesicle activation	Sinapine thiocyanate	Spontaneously hypertensive rats, HUVECs	*In vivo, In vitro*	8.54 mg kg^−1^, 50 mg L^−1^	Liu et al. (2020)
	Cardioprotective activity	Reduce oxidative stress and Ca+, anti-cardiac mitochondrial damage	Sinapic acid	Myocardial infarction rats	*In vivo*	12 mg kg^−1^	Stanely Mainzen Prince et al. (2020)
Immunity	Anti-inflammatory effect	Inhibition of NLRP3 inflammatory vesicle activation	Sinapic acid	Bone marrow-derived macrophages	*In vitro*	100–200 μM	Lee et al. (2021)
		Increase the mRNA expression levels of ZO-1, Occludin, Claudin-1 and decrease the mRNA expression levels of TLR4, NF-kB, MLCK, IL-8, IL-1β	Sinapic acid	Caco-2 cells	*In vitro*	5, 10, 15 μM	Zhang et al. (2019)
Reproductive	Anti-testicular damage activity	Reduce MDA, PC and NO levels and increase SOD and GSH- Px activity	Sinapic acid	Testicular torsion rat model	*In vivo*	10, 20 mg kg^−1^	Unsal et al. (2021)
	Anti-prostatic hyperplasia activity	Reduce foreskin gland wet weight and serum acid phosphatase activity	Sinapine, β-sitosterol and sinalbin	Prostate hyperplasia model	*In vivo*	8, 16 mg kg^−1^	Wu et al. (2003a); Wu et al. (2003b)
Tumors	Anti-cancer activity	Increase intracellular ferrous iron, lipid peroxidation and reactive oxygen species in non-small cell lung cancer cells; downregulation of SLC7A11	Sinapine	NSCLC cells, bronchial epithelial cells	*In vitro*	0–20 μM	Shao et al. (2022)
		Inhibition of anti-apoptotic factor Bcl-2, promotion of pro-apoptotic factor Bax expression	Sinapine	H22 cells	*In vitro*	LC_50_ = 53.97 μg L^−1^	Nan (2010)
		Reduce protein expression of PTGS1, PTGS2, Bcl-2, MMP-2 and MMP-9 and increase protein expression of Bax in hepatoma cells SMMC-7721	Sinapine thiocyanate	SMMC-7721 cells	*In vitro*	0–100 μM	Chen et al. (2020)
		Reduce the expression of p-AKT (S473), β-catenin, N-cadherin, Vimentin and PCNA in skin squamous carcinoma A431 and Colo-16 cells; increase the expression of E-cadherin	Sinapine thiocyanate	A431 cells, Colo-16 cells	*In vitro*	20 μM	Su et al. (2021)
		Inhibition of p-glycoprotein expression	Sinapine	Caco-2 cells	*In vitro*	0–200 μM	Guo et al. (2014)
Other	Hypoglycemic effect	Inhibition of TNF-α production	Sinapine thiocyanate	Insulin resistance model	*In vivo*	10, 30, and 90 mg kg^−1^ d^−1^	Huang et al. (2018)
	Kidney protective effect	Upregulation of PPAR-γ expression	Sinapic acid	Rat nephrotoxic model	*In vivo*	20, 40 mg kg^−1^	Singh et al. (2020)
	Anti-oxidant effect	Scavenging superoxide anion free radicals	Sinapine thiocyanate	Superoxide radicals	*In vitro*	IC_50_ = 0.135 mM	Li et al. (2012)
		Scavenging DPPH activity, scavenging hydrogen peroxide radicals and scavenging NO radicals	Sinapic acid	Human skin fibroblasts	*In vitro*	IC_50_ = 32.1 μM	Cos et al. (2002)

### 4.1 Effect on the respiratory system

#### 4.1.1 Airway allergy reduction

**FIGURE 9:**
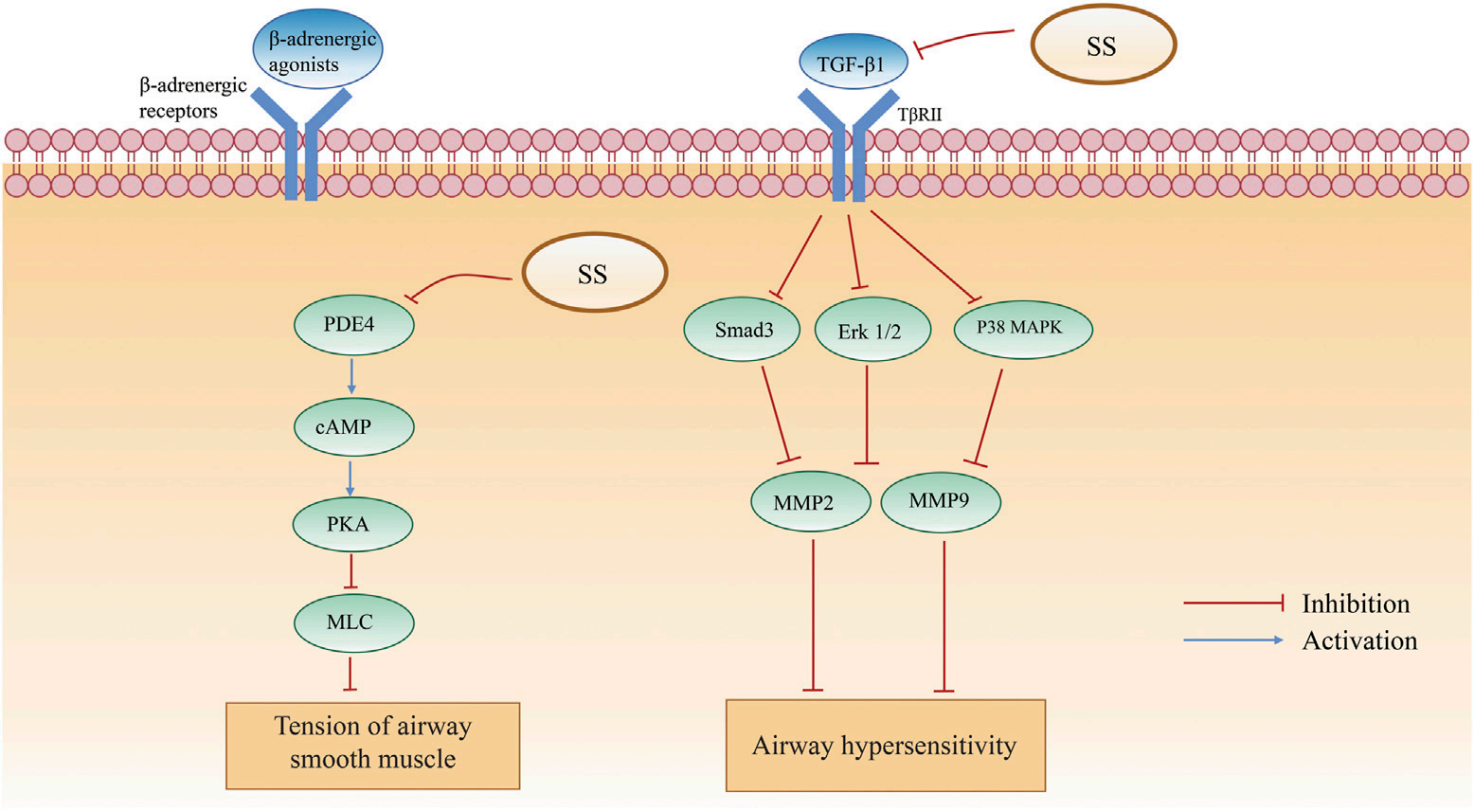
Signaling pathways of reducing airway sensitivity of SS.

Early studies have shown that asthma has three main characteristics of chronic inflammation, airway hyperresponsiveness and airway remodeling (Chen, 2016). Network pharmacology studies suggest that Peroxisome proliferator-activated receptor γ (PPARγ) and transforming growth factor-β1 (TGF-β1) are potential targets for the treatment of bronchial asthma and airway remodeling (Hu et al., 2021). SS acupoint patches can reduce airway hypersensitivity by inhibiting TGF-β and its downstream extracellular regulated protein kinases1/2 (Erk1/2) and p38 mitogen-activated protein kinase (p38 MAPK) phosphorylation to reduce matrix metalloproteinase expression and protect airway epithelial barrier-related proteins (Li, 2017). Meanwhile, SS acupoint patch can improve airway remodeling by downregulating the airway TGF-β1/Smad 3 protein expression in mice with chronic asthma, thereby treating chronic asthma (Liu et al., 2017). Inhibition of phosphodiesterase 4 (PDE4) by sinigrin lead an elevation of cAMP level and activating protein kinase A (PKA). PKA mediates a complex processes leading to an impaired ability to promote the phosphorylation of myosin light chain (MLC), leading to the relaxation of airway smooth muscle (ASM) (Chu et al., 2020). Sinapine (**1**) administered orally or as a spray could increase pulmonary and tracheal volumes by dilating airway smooth muscle, thus exerting a calming effect (Wang et al., 2011). Collectively, it can be seen that SS reduces asthma by reducing airway hypersensitivity, airway remodeling, and dilating airway smooth muscle. Herein the detailed mechanisms of airway allergy reduction induced by SS were summed in Figure 9.

#### 4.1.2 Cough suppressant inhibition

4-Hydroxybenzyl cyanide (**18**), the decomposition of which produces hydrogen cyanide, can inhibit the cough center at low doses. Studies have shown that the mechanism is to act as a central cough suppressant by inhibiting the receptors in the cough reflex arc or the vagus nerve of the afferent cough impulses (Yu, 2005). Thus, SS achieves cough suppression by inhibiting the cough suppressant.

### 4.2 Effect on the digestive system

#### 4.2.1 Hepatoprotective activity

Current studies have shown that SS is effective in nourishing the liver, improving liver fibrosis, and relieving fatty liver. On the one hand, sinapic acid (**2**) in SS inhibits bromodomain containing 4 (BRD4) expression and suppresses oxidative stress, cell scorching and hepatocyte injury (Chu et al., 2021). On the other hand, SS can prevent methotrexate (MTX)-induced liver injury by inhibiting apoptosis and stimulating Nrf2/HO-1-intermediate antioxidant enzymes through NF-κB inhibition (Ahmad et al., 2021). Sinapine (**1**) modulate the composition of intestinal flora, induce a decrease in the ratio of thick-walled phylum and mimic phylum, and increase the abundance of probiotic bacteria, thereby inhibiting hepatic steatosis (Li et al., 2019). It has also been shown that SS extract has anti-hepatic fibrosis effect and its potential mechanism of action may be related to the modulation of TGF-β1/Smad, NF-κB and AKT signaling pathways and reduction of extracellular matrix deposition (Cao et al., 2018). The studies indicated that the underlying mechanisms of the hepatoprotective activity of SS may be mediated by the regulation of TGF-β1/Smad, NF-κB and AKT signaling pathways.

#### 4.2.2 Adipocyte browning

Brown-like adipocytes have high caloric metabolism, characterized as high mitochondrial concentrations and high expression of uncoupling protein 1 (UCP1). Numerous studies have shown that brown adipose tissue not only has the function of keeping out the cold, but also burns excess fat and sugar to generate heat and prevent excess fat storage in the body. Sinapic acid (**2**) enhances the expression of peroxisome proliferator-activated receptor γ coactivator-1α (PGC-1α) and UCP1 (Bae and Kim, 2020). Thus, sinapic acid (**2**) could be used to weight loss by initiating adipocyte browning through the p38 MAPK/CREB signaling pathway.

### 4.3 Effect on neurodegenerative disorder

Sinapic acid (**2**) in SS can increase cell viability and protect cells from 6-OHDA-induced apoptotic cell death. It significantly blocks oxidative stress, including excessive production of reactive oxygen species (ROS) and decrease in the expression level of antioxidant proteins, and it also reduces mitochondrial dysfunction and endoplasmic reticulum (ER) stress, and observably inhibits mitogen-activated protein kinase (MAPK) protein activation (Zare et al., 2015; Tungalag and Yang, 2021), thereby preventing neurodegenerative diseases. Sinapic acid (**2**) also promotes CREB mRNA transcription in cells, activates the cAMP/PKA/CREB signaling pathway, improves Aβ1-42-induced PC12 cell morphology, and reduces Aβ42 content in cells (Xue et al., 2021; Xue et al., 2022). The potential therapeutic effect of sinapic acid (**2**) is due to its attenuation of KA-induced neuronal damage in the brain *via* its anti-convulsive activity through gamma-aminobutyric acid (GABA) (A) receptor activation and radical scavenging activity (Kim et al., 2010). Thus SS can be used as apotential agent to prevent neurodegenerative diseases and reduce nerve damage by improving cell damage and increasing cell viability.

### 4.4 Effect on the cardiovascular system

#### 4.4.1 Anti-hypertension

SS not only protects vascular endothelial function in SHRs by inhibiting nucleotide-binding oligomerization domain, leucine-rich repeat and pyrin domain-containing 3 (NLRP3) inflammatory vesicle activation and expression of associated inflammatory mediators, but also ameliorates AngII-induced vascular endothelial injury (Liu et al., 2020). Elevated blood pressure is accompanied by alterations in vascular endothelial cell morphology and function. Sinapine thiocyanate (**3**) in SS has a significant effect in improving blood vessel lining damage and lowering blood pressure.

#### 4.4.2 Cardioprotective activity

Oxygen radical reactions and lipid peroxidation reactions play an important role in the metabolic processes of the body, maintaining many physiological and biochemical reactions and immune responses in the body. Both increased reactive oxygen species production and calcium overload alone can lead to ischemia-reperfusion injury. The literature suggests that SS has cardioprotective effects. Sinapic acid (**2**) reduces lipid peroxidation and calcium ions and enhances the antioxidant system and mitochondrial enzymes in rat heart mitochondria (Stanely Mainzen Prince et al., 2020), thereby acting as a cardioprotective function.

### 4.5 Effect on the immune system

SS extract inhibits the protein and mRNA levels of TNF-ɑ and IL-6, thus playing an anti-inflammatory role (Xian et al., 2018). Sinapic acid (**2**) has strong anti-inflammatory activity, which is accomplished by blocking caspase-1 activation and IL-1β secretion through inhibition of NLRP3 inflammasome activation in bone marrow-derived macrophages (BMDM) (Lee et al., 2021). It can remarkably ameliorate the occurrence of Caco-2 intercellular hyperpermeability caused by LPS by increasing the mRNA expression levels of ZO-1, Occludin, Claudin-1 and decreasing the mRNA expression levels of TLR4, NF-kB, MLCK, IL-8, IL-1β (Zhang et al., 2019). Inflammation has been correlated with many chronic diseases, which provides a research basis for SS to treat many chronic diseases.

### 4.6 Effect on the reproductive system

#### 4.6.1 Anti-testicular damage activity

Sinapic acid (**2**) is the main component of SS to protect the testicles. Studies have shown that sinapic acid (**2**) markedly reduces testicle damage, oxidative stress, inflammation, cell death, and restores reduced antioxidant enzyme activity, which has a protective effect on the testicle and improves ischemia-reperfusion injury in the testicle (Unsal et al., 2021).

#### 4.6.2 Anti-prostatic hyperplasia activity

Prostatic hyperplasia is an androgen-dependent disease, and sinapine (**1**) significantly decreases the wet weight of the prostate, seminal vesicle and murine prepuce glands in mice, reduces serum acid phosphatase activity, and acts as an anti-androgen activity (Wu et al., 2003a). On this basis, a study showed that β-sitosterol (**144**), sinalbin (**5**), unsaturated fatty acids and other agents in SS act synergistically to achieve a variety of effects such as reducing capillary permeability, inhibiting fibrous tissue proliferation, reducing serum acid phosphatase activity, inhibiting 5α-reductase activity, and anti-androgen (Wu et al., 2003b), which have therapeutic and preventive effects on prostate hyperplasia.

### 4.7 Anti-tumor activity

**FIGURE 10:**
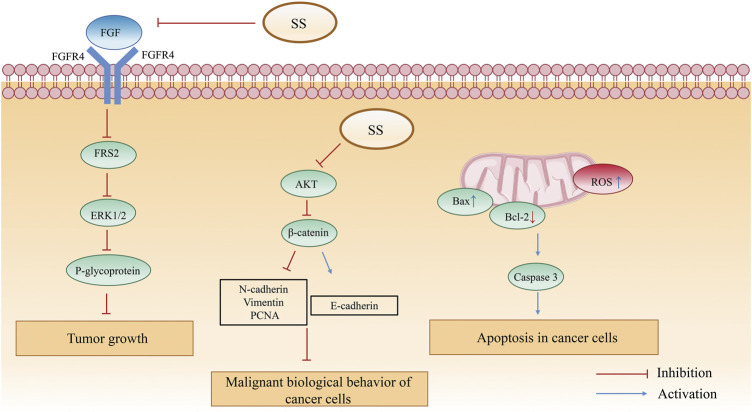
Signaling pathways of anti-tumor effect of SS.

Studies have shown that SS has clear anti-tumor effect. First of all, sinapic acid (**2**) has strong anticancer activity against various differentiated types of liver cancer cells, and in combination with cisplatin mediates the Bcl-2 assaciated X protein/B cell lymphoma/lewkmia-2 (Bax/Bcl-2) signaling pathway, upregulates LC3 protein expression, and induces hepatocellular carcinoma cell death (Zhao, 2021). And then, sinapine (**1**) restrains the proliferation of hepatocellular carcinoma H22 cells by inhibiting the anti-apoptotic factor Bcl-2 and promoting the expression of the pro-apoptotic factor Bax (Nan, 2010). It also induces cell death by increasing intracellular ferrous iron, lipid peroxidation and reactive oxygen species (ROS) in non-small cell lung cancer cells (Shao et al., 2022). Sinapine (**1**) inhibits tumor growth by suppressing the FGFR4-FRS2α-ERK1/2 signaling pathway, downregulates P-glycoprotein expression (Guo et al., 2014), antagonizes the mutagenic effect of cyclophthalamide on mouse bone marrow cells, downregulates the expression of the apoptosis suppressor gene Bcl-2 in tumor cells, and facilitates apoptosis in mouse sarcoma S180 cells (Ke, 2008). Finally, sinapine thiocyanate (**3**) from SS significantly decreases the protein expression of PTGS1, PTGS2, Bcl-2, MMP-2 and MMP-9 and increases the protein expression of Bax to prohibit the proliferation, migration and invasion of hepatocellular carcinoma cells in SMMC-7721 (Wu et al., 2003b). In addition, sinapine thiocyanate (**3**) inhibits the malignant biological behavior of skin squamous carcinoma A431 and Colo-16 cells through the AKT/β-catenin pathway (Su et al., 2021). Thus, SS achieves antitumor activity by promoting the expression of apoptotic factors and inhibiting the expression of growth factors in tumor cells. The specific mechanism of antitumor activity of SS is shown in Figure 10.

### 4.8 Others

#### 4.8.1 Hypoglycemic effect

Sinapine thiocyanate (**3**) dose-dependently decreases the levels of lipids, blood glucose, TNF-α and other metabolism-related indicators, delays hepatocyte steatosis and atherosclerosis (Huang et al., 2018). Sinalbin (**5**) achieves hypoglycemic effects by inhibiting α-glucosidase and α-amylase (Abbas et al., 2017).

#### 4.8.2 Protective effect against renal injury

Studies have shown that sinapic acid (**2**) treatment provides a dose-dependent and significant kidney protection against cisplatin-mediated nephrotoxicity in rats (Singh et al., 2020) by up-regulating PPAR-γ expression.

#### 4.8.3 Anti-oxidative activity

SS extract, sinapic acid (**2**) and sinapine thiocyanate (**3**) have been reported to have antioxidant activity (Dubie et al., 2013). Sinapine thiocyanate (**3**) functions as an antioxidant by scavenging superoxide anion radicals (Li et al., 2012). Sinapic acid (**2**), as one of phenols, has antioxidant effects by scavenging (DPPH) radicals, scavenging hydrogen peroxide radicals, scavenging NO radicals, and inhibiting microsomal lipid peroxidation activity (Cos et al., 2002; Chen, 2016). Unfortunately, the antioxidant effect of other components of SS has not been seen.

#### 4.8.4 Effect on orthopaedic diseases

Lumbar disc herniation, cervical spondylosis, scapulohumeral periarthritis, etc. are common and frequently occurring diseases. SS plays a useful role in the treatment of lumbar disc herniation, cervical spondylosis, scapulohumeral periarthritis, joint synovitis, osteoarthritis and various kinds of bi syndrome, but its mechanism of action has been poorly reported and needs further study (Zhou et al., 2014).

#### 4.8.5 Effect on wound healing

Sinapic acid (**2**) has been shown to promote the healing of chronic diabetes wounds and is used in wound dressing hydrogels with multifunctional properties. In addition, sinigrin (**4**) has therapeutic potential for wound healing activity (Mazumder et al., 2016; Liu et al., 2021; Chandika et al., 2022).

To conclude, although many of the pharmacological effects of SS and its ingredients have been confirmed, but the pharmacological effects are mainly focused on sinapine (**1**), sinapic acid (**2**) and sinapine thiocyanate (**3**). The models used in the literature are all internationally accepted models that have been summarized and displayed in Table 1. Unfortunately, the scope of pharmacological action and the depth of mechanism of action need to be further expanded. Cough relieving and asthma calming is the main pharmacological action of SS. The components and targets of action can be studied more carefully from the perspective of single compounds. In addition, it is important to emphasize that studies on the pharmacological effects and mechanisms of other compounds need to be strengthened. To date, large amount of research of SS have been focused on pharmacological activity in animal or *in vitro* cellular models, and few clinical trials have been conducted, which limits its application. Therefore, formal clinical trials should be encouraged to provide more solid evidence for application of SS.

## 5 Toxicity

The toxicity of SS is mainly characterized by skin irritation. SS is frequently used as the primary or formula application for acupuncture point compresses due to its stimulating effect on the skin. Some studies have shown that blistering of the skin occurs in almost 2 h during the application of SS *in vitro* and that the dose is positively correlated with the size of the blister (Wu et al., 2016). Interestingly, some studies have shown a significant association between the reactive symptoms of SS compresses and the therapeutic effect to be effective in patients with asthma (Ji, 2006). On the contrary, it has also been shown that there is no direct correlation between skin reactivity and efficacy and that drug-acupuncture point interrelationships have to be considered (Cui et al., 2014), the mechanism of which is unclear and needs to be investigated. Another study illustrated that the ratio of raw and cooked SS should be appropriate for topical application by comparing EOS, IgE, and IgM, and the optimal ratio of raw and cooked SS is 1:2, which has good efficacy and less adverse skin reactions (Lai et al., 2019). There is also literature comparing the skin irritation of SS from different speices, and it was found that yellow mustard seeds: white mustard seeds (5:5) for triphala paste had moderate skin irritation (Lu et al., 2020). Reducing the skin irritation of SS is promising for research development.

The skin irritation properties of SS also make it commonly used clinically as a transdermal absorption enhancer for Chinese herbal patches. The three pathways of drug transmission through the skin are intercellular transmission, intracellular transmission, and appendage transmission (Herman and Herman, 2015). The volatile oils of SS mainly composed of allyl isothiocyanate (**86**) and cyclohexyl isothiocyanate (**84**) can inhibit Ca^2+^-ATPase activity, increase intracellular Ca^2+^ concentration, alter the membrane potential of Ha CaT cells, and promote drug entry into the skin (Ruan et al., 2019). Topical application of SS extract also induced morphological changes in langerhans cells in the skin and stimulated the secretion of IL1β and TNFα in the skin, suggesting activation of the immune response (Guo et al., 2013).

In short, volatile oils are believed the main component that causes skin irritation. The factors leading to skin irritation may be related to the time of patching, the method of concoction, and the original plant. The mechanism of skin irritation and the correlation between skin reaction and drug efficacy deserve further study. Reducing skin irritation is of great importance in the clinical application of SS. However, it is worth noting that the above evaluation methods for the severity of skin irritation are subjective. Therefore, it is particularly important to establish a more objective and recognized evaluation method for skin irritation.

## 6 Analytical methods

### 6.1 Compositional analysis of medicinal materials

The method of GC-MS is usually used for thermally stable samples. The volatile oils and fatty acid compounds from SS have been characterized using GC-MS. The extract method of steam distillation was used to extract volatile oil from SS. The volatile oils of SS were analyzed by GC-MS and were qualitative by NIST database (Liu et al., 2007; Wu et al., 2010). The fatty acid components of SS were extracted by petroleum ether and the methyl ester reagents were added for methylation. Then the liquid-liquid extractions were applied to extract the ingredients for analysis by GC-MS (Shi et al., 2003; Zhang and Wang, 2006; Li and Lin, 2012). The method of headspace solid phase micro-extraction (HS-SPME) was used to extract volatile components from SS. The volatile components were analyzed and identified by GC-MS (Cai et al., 2014). Confirmation of the volatile chemical structures of SS should be achieved unambiguously using authentic standards or publications (measured under identical conditions), if no authentic standards are available (Indrayanto, 2022). However, the extraction methods and GC-MS analysis conditions mentioned above were different. The MS data were also only searched by the NIST database and the results are difficult to be repeated by subsequent researchers.

Zhang et al. (2021) established a method for content determination of sinapine thiocyanate (**3**) by HPLC-UV. The methodological validation included linearity, precision, repeatability, stability and recovery were conformed with Chinese Pharmacopoeia (CP). The similarity of 13 batches of SS fingerprint was more than 0.943. Similarly, He et al. (2020) established the UPLC-UV fingerprints of SS and its formulation granule. The similarities of 15 batches of standard decoction or formulation granule were greater than 0.99. The available authentic standard sinapine thiocyanate (**3**) was used as the reference peak to calculate the relative retention time of other common peaks in the above fingerprint methods. It was very crucial for quality assessment of using chemical profiling.

UPLC-Q-Exactive Obitrap MS was adopted to analyze chemical constituents of SS before and after stir-frying. The chemical constituents of SS before and after stir-frying were identified by Compound Discover 3.2 software combined with *m/z* Cloud database, high resolution MS OTCML database or compared with the available authentic standards (Jia et al., 2021). The chemometric analysis was not methodologically validated and the stability of the QC samples was not evaluated (ORA Laboratory Manual, 2022). The chemometrics method only was performed for the content changes of chemical constituents using peak area as variate (Jia et al., 2021). Popova et al. established a simple and fast ion chromatography (IC) method for the simultaneous quantification of sinigrin (**4**), sinalbin (**5**), and anionic hydrolysis products of SS. The related compounds were purchased from Sigma-Aldrich (St. Louis, MO, United States) or isolated in their laboratory. Calibration curve, limits of detection (LOD), and limits of quantification (LOQ) for two intact glucosinolates (sinigrin and sinalbin) and anionic hydrolysis products (SO_4_^2−^ and SCN^−^) were determined by the proposed IC method (Popova and Morra, 2014). Nicácio et al. (2021) optimized the QuEChERS extraction method followed by UHPLC-MS/MS analysis for phenolic compounds determination in three species of SS. 21 phenolic compounds were estimated, and the linearity, LOD and LOQ were determined. The intra-day, and inter-day precisions were carried out and expressed in terms of relative standard deviation (RSD). Rochfort et al. (2008) established a method of ion trap mass spectrometry for glucosinolates. This method takes advantage of the glucosinolate anion fragmentation which consistently produces a sulphonate ring-opened glucose moiety (*m/z* 259) in the ion trap mass spectrometer. The strategy could be applied to determine glucosinolates in SS.

Determination of the chemical structure of the unknown compounds could be performed by using complete spectroscopic analysis (UV-VIS, IR, CD, HR-MS and NMR). SS was extracted and partitioned and the ten chemical constituents were purified by chromatography and recrystallization and then the structures were determined by nuclear magnetic resonance (^1^H-NMR, ^13^C-NMR, HMQC and HMBC) (Feng et al., 2008).

In a number of quantitative cases, authors only selected validation parameters (LOD, LOQ, linearity, recovery, precision, stability and matrix effect, etc.) which were under close interest and not all parameters were investigated. In addition, some validation parameters also did not conform to the published guidelines (Kruve et al., 2015). The confirmation of peak identity and quantitative methods validation parameters were described in detail in Table 2.

**TABLE 2:** Analytical method of SS.

Classification	Analytical method	Constituents	Confirmation of peak identity	Methods validation parameters	References
Compositional analysis of medicinal materialsI	GC-MS	44 Components in the volatile oil of SS	Not confirmed	/	Liu et al. (2007)
	GC-MS	7 Components in the volatile oil of SS	Not confirmed	/	Wu et al. (2010)
	GC-MS	6 Fatty acids and 1 alkane	Not confirmed	/	Shi et al. (2003)
	GC-MS	18 Fatty acids and 5 non-fatty acid components	Not confirmed	/	Li and Lin (2012)
	GC-MS	15 Fatty acids and 1 unsaturated alcohol	Not confirmed	/	Zhang and Wang (2006)
	HS-SPME-GC-MS	25 Volatile components	Not confirmed	/	Cai et al. (2014)
	HPLC-UV	Sinapine thiocyanate (**3**)	Confirmed	Linearity: 25–400 μg/mL; RSD of precision: 0.14%; RSD of repeatability: 0.12%; RSD of stability: 0.04%; Recovery: 102.1%; Similarity evaluations: *r* close to 1	Zhang et al. (2021)
	UPLC-UV	Sinapine (**1**) and sinapic acid (**2**)	Confirmed	RSD of precision < 3%; RSD of stability < 3%; RSD of repeatability < 3%; Similarity evaluations: *r* close to 1	He et al. (2020)
	UPLC-Q-Exactive Obitrap MS	54 Chemical constituents, mainly fatty acids [represented by sinapic acid (**2**)], alkaloids [represented by sinapine (**1**)], flavonoids and other compounds	Confirmed	Five components [represented by sinapine (**1**)] were compared to the Standards; Remaining components: accurate mass accuracy ppm < 5; RSD of retention times (Rt) < 5%; Chemometric analysis was not methodologically validated	Jia et al. (2021)
	Ion chromatography	Sinigrin (**4**), sinalbin (**5**)	Confirmed	Sinigrin (**4**): linearity: 0.03–2.0 mM; Recovery: 85%–102%; RSD of reproducibility: 1.1%–2.4%; RSD of stability: <3%; RSD of precision: 2%–9% Sinalbin (**5**): linearity: 0.01–2.0 mM; Recovery: 95%–98%; RSD of reproducibility: 1.0%–1.9%; RSD of stability: <3%; RSD of precision: 2%–6%	Popova and Morra (2014)
	QuEChERS-UHPLC-MS/MS	21 Phenolic compounds	Confirmed	Linearity (r) > 0.99; RSD of precision: 18.5%	Nicácio et al. (2021)
	Ion trap mass spectrometry	6 Glucosinolates	Confirmed	Linearity (R^2^): 0.9972-0.9998; CV of reproducibility: 5%	Rochfort et al. (2008)
	NMR	Sinapic acid (**2**), 4-hydroxybenzoic acid (**19**), 4-Hydroxybenzaldehyde (**20**), etc.	Confirmed	^1^H-NMR, ^13^C-NMR, HMQC, HMBC	Feng et al. (2008)
Biological sample analysis	Paper Chromatography	Metabolic pathways of sinapic acid (**2**)	Not confirmed	/	Griffiths (1969)
	UPLC-MS/MS	Pharmacokinetics of sinapic acid (**2**)	Confirmed	Linearity: 10–5000 μg/L (R^2^ = 0.9995); RSD of precision: 1.1%–4.1%; Accuracy: 99.1%–107.3%; Recovery: 95.6%–107.5%; Matrix effect: 90.3%–103.2%; RSD of stability < 3.7%	Li et al. (2020)
	UPLC-MS/MS	Tissue distribution of sinapine thiocyanate (**3**)	Confirmed	Linearity: 0.2–112.5 ng/mL (r = 0.9948); LLOQ: 0.2 ng/mL; Matrix effect: 83.0%–115.15%; Recovery: 44.77%–145.19%; RSD of precision: 2.74%–10.38%; RSD of stability: 1.78%–10.15%	Tang et al. (2022)
	UPLC-Q/TOF-MS	Metabolic pathways of sinapine thiocyanate (**3**)	Confirmed	Two ions (parent ion and product ion) with accurate mass accuracy < 5 ppm	Guan et al. (2022)

Note: /, the data is not described in the literature.

### 6.2 Biological sample analysis

The metabolism of sinapic acid (**2**) in rats has been studied by researchers as early as 1969 using paper chromatography (Griffiths, 1969), and a metabolic pathway of sinapic acid (**2**) was proposed. Due to the limitations of the technique, the metabolites of sinapic acid (**2**) were compared with reference materials prepared in laboratory only by the maximum ultraviolet absorption and Rf values. Because of the complexity of metabolites *in vivo*, it was easy to cause false positive results. In recent years, the pharmacokinetics of sinapic acid (**2**) from SS in rat plasma has been investigated by a validated UPLC-MS/MS method (Li et al., 2020). The dynamic distribution of sinapine thiocyanate (**3**) has been monitored using a validated UPLC-MS/MS method (Tang et al., 2022). The quantitative method validation was established according to the United States Food and Drug Administration (FDA) guidelines (US Food and Drug Administration, 2018). The items of validation included selectivity, linearity, precision, accuracy, matrix effect, extraction recovery, and stability. In addition, the metabolic pathways of sinapine thiocyanate (**3**) have also been studied using UHPLC-Q/TOF-MS in rat plasma, urine and fecal samples after oral administration of sinapine thiocyanate (**3**) (Guan et al., 2022). Thirteen metabolites were structurally identified, and the proposed metabolic pathways of sinapine thiocyanate (**3**) included deamination, demethylation, hydrogenation, dehydration, and extensive conjugation. Methods validation parameters of biological samples were also summarized in Table 2. The assay of mass spectrometry, especially combined mass spectrometry, will be a powerful analytical tool for determination of trace metabolites *in vivo* in the pharmacokinetic and metabolic study of SS.

## 7 Pharmacokinetics

In the last decades, the active ingredients in SS have been analyzed *in vivo* and *in vitro* to study their biotransformation profiles. These studies focused on glucosinolates and their metabolites, such as sinalbin (**5**), sinapic acid (**2**), sinapine thiocyanate (**3**), and thioglucoside analogues. Glucosinolates are hydrolyzed by the mustardase to produce p-hydroxybenzyl isothiocynate (white mustard oil), sinapine bisulfate (acidic mustard base) and glucose. Sinapine bisulfate is hydrolyzed by alkaline hydrolysis to produce sinapic acid (**2**) and choline. The p-hydroxybenzyl isothiocyanate is extremely unstable in alkaline solution and decomposes into the p-hydroxybenzyl alcohol and thiocyanate (Wan, 2014). In addition, administration of sinapic acid (**2**) to rats results in urinary excretion of 3-hydroxy-5-methoxyphenylpropionic acid, dihydro sinapic acid (**2**), 3-hydroxy-5-methoxycinnamic acid, and unchanged sinapic acid (**2**). The sinapine (**1**) is also catabolized to free sinapic acid (**2**) and 3-hydroxy-5-methoxyphenylpropionic acid in rats. 3,4,5-Trimethoxycinnamic acid is partially metabolized to sinapic acid (**2**) and 3-hydroxy-5-methoxyphenylpropionic acid. And then 3,5-dimethoxycinnamic acid is metabolized to 3-hydroxy-5-methoxycinnamic acid and 3-hydroxy-5-methoxyphenylpropionic acid (Griffiths, 1969). Two proteins (CFPTT and CfPbgS) have also been shown to be the enzymes responsible for the degradation of sinalbin (**5**). Sinalbin (**5**) is absorbed and phosphorylated by CfPttS and subsequently, the phosphorylated entity is degraded by CfPbgS (Watanabe et al., 2021). Metabonomics studies have shown that the extensive metabolism of sinapine thiocyanate (**3**) includes deamination, demethylation, reduction, dehydration, glucuronide incorporation and sulfate incorporation to produce 11 metabolites (Guan et al., 2022).

Previous pharmacokinetics focused on sinapine thiocyanate (**3**), sinapic acid (**2**), and sinapine chloride. The pharmacokinetic parameters of sinapine thiocyanate (**3**) after intravenous (2 mg/kg) and intragastric (100 mg/kg) administrations were obtained. The parameter values of T_max_ and C_max_ were 88.74 ± 20.08 min and 47.82 ± 18.77 nM, respectively. The T_1/2_ of 67.52 ± 15.69 min in the oral administration group was lower than it in the intravenous administration group (Guan et al., 2022). There are studies in the literature showing that the area under the drug-time curve (AUC_0-t_), mean retention time (MRT_0-t_), of different dose groups of sinapic acid (**2**) AUC_0-t_ *in vivo* showed a good linear dependence between 4.5, 9 and 18 g/kg, and the pharmacokinetic curve of sinapic acid (**2**) showed a double peak with a low front and a high back (Li et al., 2020). The cumulative absorption and metabolism rates of sinapine chloride in the intestinal sac at 90 min reached (5.78 ± 1.39)% and (9.42 ± 1.97)%. Sinapine chloride is rapidly absorbed after gavage administration, reaching peak blood concentrations at about 2 h; however, it is also rapidly metabolized in the serum, reaching its half-life at about 3 h (Wang et al., 2020).

From the pharmacokinetics and bioavailability of sinapine thiocyanate (**3**), sinapic acid (**2**), and sinapine chloride, it can be concluded that the bioavailability of SS is low due to enterohepatic circulation. The study of the *in vivo* absorption, distribution, metabolism, and excretion process of other ingredients in SS is also significant and deserve further study. It is extremely important to study its percutaneous process and pharmacokinetics for external use in order to provide support for the mechanism study of topical treatment of SS in various skin diseases or systemic disease, and the safety evaluation of skin irritants.

## 8 Conclusion and perspectives

Taken together, due to its extensive ethnomedicine uses reported for thousands of years, SS has become one of the most important components of many traditional Chinese medicine and ethnic prescriptions. Clinical applications of synergistic compounding methods have shown that SS has therapeutic effects on asthma, bronchitis, tendon pain, prostate enlargement, hyperlipidemia, hyperglycemia, hypertension, tumors, and cancer. SS has been made into paste, patch, decoction, powder, pill, and medicinal liquor. According to the current literatures, glucosinolates are hydrolyzed by enzymes to produce sinapine (**1**), sinapic acid (**2**), sinapine thiocyanate (**3**), and isothiocyanates. They have significant anti-cough and asthma, anti-inflammatory, anti-nerve damage, anti-androgenic effects, cardioprotective, anti-tumor effects and pro-skin penetration. SS is mostly used topically with other drugs, but clinical practice has shown that they have severe skin irritation and can cause allergic contact dermatitis. It has been documented that SS produces its effects through this skin irritation. Although significant breakthroughs have been made in the comprehensive exploration and application of SS, there is still some in-depth work to be done in the future.

Firstly, glucosinolates are important components in SS. Due to the high hydrophilicity and structural similarity, its purification, isolation and analytical methods need further study. Meanwhile, the hydrolysis process of glucosinolates can also be a future research direction. Secondly, although SS has many pharmacological activities, they are mainly focused on sinapine (**1**), sinapic acid (**2**), sinapine thiocyanate (**3**). The pharmacological effects of other components can be further studied, and the molecular targets of many pharmacological effects are not yet clear. Thirdly, reducing the skin irritation of SS is essential for its clinical application. There are fewer studies on the relationship between irritation, efficacy and chemical composition. Further studies especially those focused on the appropriate dose, efficacy, and safety of SS and its metabolites are recommended before subjecting SS to clinical trials. Finally, the absorption and metabolism studies of SS have focused on sinapine thiocyanate (**3**) and sinalbin (**5**) after oral administration, but pharmacokinetic studies of the components in SS after topical administration are limited. Therefore, elucidating the pharmacokinetic properties of the active components of SS after topical administration may be a valuable research direction.

## Abbreviations

Bax/Bcl-2, Bcl-2 assaciated X protein/B cell lymphoma/lewkmia-2; BMDM, Bone marrow-derived macrophages; BRD4, Bromodomain containing 4; CP, Chinese Pharmacopoeia; ESI, Electrospray ionization; ER, Endoplasmic reticulum; Erk1/2, Extracellular regulated protein kinases1/2; GABA, Gamma-aminobutyric acid; GC-MS, Gas chromatograph-mass spectrometer; HS-SPME, Headspace solid phase micro-extraction; IC, Ion chromatopraphy; LOD, Limits of detection; LOQ, Limits of quantification; MTX, Methotrexate; NLRP3, Nucleotide-binding oligomerization domain, leucine-rich repeat and pyrin domain-containing 3; NMR, Nuclear magnetic resonance spectroscopy; P38 MAPK, P38 mitogen-activated protein kinase; PGC-1α, Peroxisome proliferator-activated receptor γ coactivator-1α; PPARγ, Peroxisome proliferator-activated receptor γ; QuEChERS, Quick, Easy, Cheap, Effective, Rugged, Safe; ROS, Reactive oxygen species; RSD, Relative standard deviation; SS, Sinapis Semen; TGF-β1, Transforming growth factor-β1; UCP1, Uncoupling protein 1; UHPLC-MS/MS, Ultra performance liquid chromatography/tandem mass spectrometry.
