# Supplementary material for: Sinapis Semen: A review on phytochemistry, pharmacology, toxicity, analytical methods and pharmacokinetics
Source: Front Pharmacol. 2023 Apr 12;14:1113583. doi: 10.3389/fphar.2023.1113583 (PMC10130658; doi:10.3389/fphar.2023.1113583)
Supplement: Supplementary file 1 [file Table1.doc]

**Supplementary material**

**Sinapis Semen: A review on phytochemistry, pharmacology, toxicity, analytical methods and pharmacokinetics**

Rui Dang^1^, Huida Guan^1^, Changhong Wang*

*Institute of Chinese Materia Medica, Shanghai University of Traditional Chinese Medicine, The MOE Key Laboratory for Standardization of Chinese Medicines, Shanghai R&D Centre for Standardization of Chinese Medicines, 1200 Cailun Road, Shanghai, 201203, China.*

*Corresponding author: Professor Changhong Wang. The Institute of Chinese Materia Medica, Shanghai University of Traditional Chinese Medicine, Shanghai, 201203, China.

Tel.: +86-021-51322511.

E-mail: [wchcxm@hotmail.com](mailto:wchcxm@hotmail.com); [wchcxm@shutcm.edu.cn](mailto:wchcxm@shutcm.edu.cn)

^1^Rui Dang and Huida Guan contributed equally to this work

**Table S1. Chemical components identified from SS**

| No. | Classification | Name | Formula | molecular weight | *S. alba* | *B. juncea* | Reference | |  |
| --- | --- | --- | --- | --- | --- | --- | --- | --- | --- |
| 1 | Glucosinolates and their hydrolysates | Sinapine | C_16_H_24_NO_5_^+^ | 310.37 | + |  | Zhang et al. (2015) | |  |
| 2 | Glucosinolates and their hydrolysates | Sinapic acid | C_11_H_12_O_5_ | 224.21 | + |  | Zhang et al. (2015) | |  |
| 3 | Glucosinolates and their hydrolysates | Sinapine thiocyanate | C_17_H_24_N_2_O_5_S | 368.45 | + | + | Zhang et al. (2015) | |  |
| 4 | Glucosinolates and their hydrolysates | Sinigrin | C_10_H_17_NO_9_S_2_ | 359.40 |  | + | Zhang et al. (2010); Zhang et al. (2015) | |  |
| 5 | Glucosinolates and their hydrolysates | Sinalbin | C_14_H_19_NO_10_S_2_ | 425.43 | + |  | Zhang et al. (2015) | |  |
| 6 | Glucosinolates and their hydrolysates | Gluconapin | C_11_H_19_NO_9_S_2_ | 373.40 | + |  | Zhang et al. (2015) | |  |
| 7 | Glucosinolates and their hydrolysates | Progoitrin | C_11_H_19_NO_10_S_2_ | 389.40 | + |  | Zhang et al. (2015) | |  |
| 8 | Glucosinolates and their hydrolysates | Glucoibervirin | C_11_H_21_NO_9_S_3_ | 407.50 | + |  | Zhang et al. (2015) | |  |
| 9 | Glucosinolates and their hydrolysates | Glucoiberin | C_11_H_21_NO_10_S_3_ | 423.48 | + |  | Zhang et al. (2015) | |  |
| 10 | Glucosinolates and their hydrolysates | Glucocheirolin | C_11_H_21_NO_11_S_3_ | 439.50 | + |  | Zhang et al. (2015) | |  |
| 11 | Glucosinolates and their hydrolysates | 8- (Methylsulfanyl) octyl-glucosinolate | C_16_H_31_NO_11_S_3_ | 509.60 | + |  | Zhang et al. (2015) | |  |
| 12 | Glucosinolates and their hydrolysates | Nonyl glucosinolate | C_16_H_31_NO_9_S_2_ | 445.60 | + |  | Zhang et al. (2015) | |  |
| 13 | Glucosinolates and their hydrolysates | Gluconasturtiin | C_15_H_21_NO_9_S_2_ | 423.46 | + |  | Zhang et al. (2015) | |  |
| 14 | Glucosinolates and their hydrolysates | Glucobrassicin | C_16_H_20_N_2_O_9_S_2_ | 448.47 | + |  | Zhang et al. (2015) | |  |
| 15 | Glucosinolates and their hydrolysates | 4-Hydroxybenzylglucosinolate | C_14_H_19_NO_10_S_2_ | 425.42 | + |  | Zhang et al. (2015) | |  |
| 16 | Glucosinolates and their hydrolysates | 4-Hydroxyglucobrassicin | C_16_H_20_N_2_O_10_S_2_ | 464.47 | + |  | Zhang et al. (2015) | |  |
| 17 | Glucosinolates and their hydrolysates | 4-Hydroxybenzoic acid | C_7_H_6_O_3_ | 138.12 | + |  | Yu (2005) | |  |
| 18 | Glucosinolates and their hydrolysates | 4-Hydroxybenzyl cyanide | C_8_H_7_NO | 173.17 | + |  | Yu (2005) | |  |
| 19 | Glucosinolates and their hydrolysates | 4-Hydroxyphenylacetic acid | C_8_H_8_O_3_ | 152.15 | + |  | Yu (2005) | |  |
| 20 | Glucosinolates and their hydrolysates | 4-Hydroxybenzaldehyde | C_7_H_6_O_2_ | 122.12 | + |  | Yu (2005) | |  |
| 21 | Glucosinolates and their hydrolysates | 4-Hydroxybenzoylcholine | C_12_H_18_NO_3_^+^ | 224.28 | + |  | Yu (2005) | |  |
| 22 | Glucosinolates and their hydrolysates | 3-Hydroxy-4-methoxycinnamoylcholine | C_15_H_22_NO_4_^+^ | 280.34 | + |  | Zhang et al. (2015) | |  |
| 23 | Glucosinolates and their hydrolysates | 3, 4-Methoxybenzoyl choline | C_14_H_22_NO_4_^+^ | 268.33 | + |  | Zhang et al. (2015) | |  |
| 24 | Volatile oil | Acrylonitrile | C_3_H_3_N | 53.06 | + | + | Liu et al. (2007); Zhang et al. (2007) | |  |
| 25 | Volatile oil | Crotononitrile | C_4_H_5_N | 67.09 | + |  | Liu et al. (2007) | |  |
| 26 | Volatile oil | Allyl cyanide | C_4_H_5_N | 67.09 | + |  | Wu et al. (2010) | |  |
| 27 | Volatile oil | 3-Chloropropiononitrile | C_3_H_4_ClN | 89.52 | + | + | Liu et al. (2007); Zhang et al. (2007) | | |
| 28 | Volatile oil | 4-Pentenenitrile | C_5_H_7_N | 81.12 |  | + | Zhang et al. (2007) | | |
| 29 | Volatile oil | Benzenepropanenitrile | C_9_H_9_N | 131.17 | + | + | Zhang et al. (2007) | | |
| 30 | Volatile oil | 3-Methylcrotononitrile | C_5_H_7_N | 81.12 | + |  | Wu et al. (2010) | | |
| 31 | Volatile oil | Hexanenitrile | C_6_H_11_N | 97.16 | + | + | Liu et al. (2007); Zhang et al. (2007) | |  |
| 32 | Volatile oil | Valeronitrile | C_5_H_9_N | 83.13 | + | + | Liu et al. (2007); Zhang et al. (2007) | |  |
| 33 | Volatile oil | Glyceraldehyde | C_3_H_6_O_3_ | 90.08 | + |  | Wu et al. (2010) | |  |
| 34 | Volatile oil | 2-Ethoxypropane | C_5_H_12_O | 88.15 | + |  | Wu et al. (2010) | |  |
| 35 | Volatile oil | β- Selinene | C_15_H_24_ | 204.35 | + |  | Liu et al. (2007) | |  |
| 36 | Volatile oil | Caryophyllene | C_15_H_24_ | 204.35 | + | + | Chen et al. (2006); Zhang et al. (2007) | |  |
| 37 | Volatile oil | 2-Methyl-1-pentene | C_6_H_12_ | 84.16 | + |  | Liu et al. (2007) | |  |
| 38 | Volatile oil | 2-Heptanol | C_7_H_16_O | 116.20 | + |  | Liu et al. (2007) | |  |
| 39 | Volatile oil | 2-Pentylfuran | C_9_H_14_O | 138.21 | + | + | Liu et al. (2007); Zhang et al. (2007) | |  |
| 40 | Volatile oil | 2,4-Dodecadien-1-al | C_12_H_20_O | 180.29 | + |  | Liu et al. (2007) | |  |
| 41 | Volatile oil | 2,4-Heptadienal | C_7_H_10_O | 110.15 | + |  | Liu et al. (2007) | |  |
| 42 | Volatile oil | Phenylacetaldehyde | C_8_H_8_O | 120.15 | + | + | Liu et al. (2007); Zhang et al. (2007) | |  |
| 43 | Volatile oil | 1-Octanol | C_8_H_18_O | 130.23 | + |  | Liu et al. (2007) | |  |
| 44 | Volatile oil | 2,3-Butanedione | C_4_H_6_O_2_ | 86.09 | + | + | Liu et al. (2007); Zhang et al. (2007) | |  |
| 45 | Volatile oil | 2,3-Pentanedione | C_5_H_8_O_2_ | 100.12 | + | + | Liu et al. (2007); Zhang et al. (2007) | |  |
| 46 | Volatile oil | 3-Octen-2-one | C_8_H_14_O | 126.20 | + |  | Liu et al. (2007) | |  |
| 47 | Volatile oil | 3,5-Octadien-2-one | C_8_H_12_O | 124.18 | + |  | Liu et al. (2007) | |  |
| 48 | Volatile oil | Cyclohexanone | C_6_H_10_O | 98.14 |  | + | Zhang et al. (2007) | |  |
| 49 | Volatile oil | 2-Methyl-undecanal | C_12_H_24_O | 184.32 | + |  | Liu et al. (2007) |  | |
| 50 | Volatile oil | Naphthalene | C_10_H_8_ | 128.17 | + |  | Liu et al. (2007) |  | |
| 51 | Volatile oil | 2-Ethylnaphthalene | C_12_H_12_ | 156.22 | + |  | Liu et al. (2007) |  | |
| 52 | Volatile oil | 2-Nonenal | C_9_H_16_O | 140.23 | + |  | Liu et al. (2007) |  | |
| 53 | Volatile oil | Hexanal | C_6_H_12_O | 100.16 | + | + | Liu et al. (2007); Zhang et al. (2007) |  | |
| 54 | Volatile oil | Nonanal | C_9_H_18_O | 142.24 | + | + | Liu et al. (2007); Zhang et al. (2007) |  | |
| 55 | Volatile oil | Octanal | C_8_H_16_O | 128.21 | + |  | Liu et al. (2007) |  | |
| 56 | Volatile oil | Furfural | C_5_H_4_O_2_ | 96.08 | + |  | Liu et al. (2007) |  | |
| 57 | Volatile oil | 5-Methylfurfural | C_6_H_6_O_2_ | 110.11 |  | + | Zhang et al. (2007) | |  |
| 58 | Volatile oil | 2-Methylthiomethyl furan | C_6_H_8_OS | 128.19 | + | + | Zhang et al. (2007) | |  |
| 59 | Volatile oil | 2-Hexylthiophene | C_10_H_16_S | 168.30 | + | + | Zhang et al. (2007) | |  |
| 60 | Volatile oil | 5,10-Pentacarbiene-1-ol | C_15_H_28_O | 224.39 | + |  | Zhang and Wang. (2006) |  | |
| 61 | Volatile oil | 2-Dodecen-1-ol | C_12_H_24_O | 184.32 | + |  | Liu et al. (2007) |  | |
| 62 | Volatile oil | Allicin | C_6_H_10_OS_2_ | 162.27 | + | + | Liu et al. (2007); Zhang et al. (2007) |  | |
| 63 | Volatile oil | 1-Pentanol | C_5_H_12_O | 88.15 | + |  | Cai et al. (2014) |  | |
| 64 | Volatile oil | 4-Terpineol | C_10_H_18_O | 154.25 | + |  | Yu (2005) |  | |
| 65 | Volatile oil | Propylcyclopropane | C_6_H_12_ | 84.16 | + |  | Cai et al. (2014) | |  |
| 66 | Volatile oil | Heptanol | C_7_H_16_O | 116.20 | + |  | Cai et al. (2014) | |  |
| 67 | Volatile oil | Eugenol | C_10_H_12_O_2_ | 164.20 |  | + | Zhang et al. (2007) | | |
| 68 | Volatile oil | 6-Methyl-5-hepten-2-one | C_8_H_14_O | 126.20 | + |  | Cai et al. (2014) | |  |
| 69 | Volatile oil | Dodecane | C_12_H_26_ | 170.34 | + |  | Cai et al. (2014) | |  |
| 70 | Volatile oil | Tridecane | C_13_H_28_ | 184.36 | + | + | Cai et al. (2014) | |  |
| 71 | Volatile oil | Tetradecane | C_14_H_30_ | 198.39 | + |  | Cai et al. (2014) | |  |
| 72 | Volatile oil | Pentadecane | C_15_H_32_ | 212.41 |  | + | Zhang et al. (2007) | | |
| 73 | Volatile oil | Cis-anethol | C_10_H_12_O | 148.20 | + |  | Cai et al. (2014) | | |
| 74 | Volatile oil | Benzothiazole | C_7_H_5_NS | 135.19 | + |  | Cai et al. (2014) | |  |
| 75 | Volatile oil | β-Patchoulene | C_15_H_24_ | 204.35 | + |  | Cai et al. (2014) | |  |
| 76 | Volatile oil | 2, 6 -Dimethylnaphthalene | C_12_H_12_ | 156.22 | + |  | Cai et al. (2014) | |  |
| 77 | Volatile oil | Butylated hydroxytoluene | C_15_H_24_O | 220.35 | + |  | Cai et al. (2014) | |  |
| 78 | Volatile oil | 1,1,6,7,10-Pentamethyl-decahydronaphthalene | C_15_H_28_ | 208.38 | + |  | Cai et al. (2014) | |  |
| 79 | Volatile oil | Cedrol | C_15_H_26_O | 222.37 | + |  | Cai et al. (2014) | |  |
| 80 | Volatile oil | ( + )- Limonene | C_10_H_16_ | 136.23 | + |  | Cai et al. (2014) | |  |
| 81 | Volatile oil | 1-Isocyanato-3-methylbutane | C_6_H_11_NO | 113.16 | + |  | Wu et al. (2010) | |  |
| 82 | Volatile oil | Phenyl isothiocyanate | C_7_H_5_NS | 135.18 | + |  | Wu et al. (2010) | |  |
| 83 | Volatile oil | 3-Butenyl isothiocyanate | C_5_H_7_NS | 113.18 | + |  | Wu et al. (2010) | |  |
| 84 | Volatile oil | Cyclohexyl isothiocyanate | C_7_H_11_NS | 141.24 | + |  | Cai et al. (2014) | |  |
| 85 | Volatile oil | Butyl isothiocyanate | C_5_H_9_NS | 115.20 | + |  | Wu et al. (2010) | |  |
| 86 | Volatile oil | Allyl isothiocyanate | C_4_H_5_NS | 99.15 | + | + | Cai et al. (2014); Zhang et al. (2007) | |  |
| 87 | Fatty acid | Oleic acid | C_18_H_34_O_2_ | 282.47 | + |  | Zhang and Wang (2006) | |  |
| 88 | Fatty acid | Palmitoleic acid | C_16_H_30_O_2_ | 254.41 | + |  | Zhang and Wang (2006) | |  |
| 89 | Fatty acid | Palmitic acid | C_16_H_32_O_2_ | 256.42 | + |  | Zhang and Wang (2006) | |  |
| 90 | Fatty acid | Linoleic acid | C_18_H_32_O_2_ | 280.45 | + |  | Zhang and Wang (2006) | |  |
| 91 | Fatty acid | γ- Linolenic acid | C_18_H_30_O_2_ | 278.44 | + |  | Zhang and Wang (2006) | |  |
| 92 | Fatty acid | Linolenic acid | C_18_H_30_O_2_ | 278.430 | + |  | Zhang and Wang (2006) | |  |
| 93 | Fatty acid | Stearic acid | C_18_H_36_O_2_ | 284.48 | + |  | Zhang and Wang (2006) | |  |
| 94 | Fatty acid | 13,11-Eicosenoic acid | C_20_H_36_O_2_ | 308.50 | + |  | Zhang and Wang (2006) | |  |
| 95 | Fatty acid | 11-Eicosenoic acid | C_20_H_38_O_2_ | 310.52 | + |  | Zhang and Wang (2006) | |  |
| 96 | Fatty acid | Arachidic acid | C_20_H_40_O_2_ | 312.53 | + |  | Zhang and Wang (2006) | |  |
| 97 | Fatty acid | Erucic acid | C_22_H_42_O_2_ | 338.57 | + |  | Zhang and Wang (2006) | |  |
| 98 | Fatty acid | Behenic acid | C_22_H_44_O_2_ | 112.13 | + |  | Zhang and Wang (2006) | |  |
| 99 | Fatty acid | 15-Tetracosenoic acid | C_24_H_46_O_2_ | 366.62 | + |  | Zhang and Wang (2006) | |  |
| 100 | Fatty acid | Lignoceric acid | C_24_H_48_O_2_ | 368.63 | + |  | Zhang and Wang (2006) | |  |
| 101 | Fatty acid | Myristic acid | C_14_H_28_O_2_ | 228.37 | + |  | Zhang and Wang (2006) | |  |
| 102 | Fatty acid | Docosadienoic acid | C_22_H_40_O_2_ | 336.55 | + |  | Zhang and Wang (2006) | |  |
| 103 | Fatty acid | Lauric acid | C_12_H_24_O_2_ | 200.32 | + |  | Zhang and Wang (2006) | |  |
| 104 | Fatty acid | Pentadecanoic acid | C_15_H_30_O_2_ | 242.40 | + |  | Zhang and Wang (2006) | |  |
| 105 | Fatty acid | Nonadecanoic acid | C_19_H_38_O_2_ | 298.50 | + |  | Zhang and Wang (2006) | |  |
| 106 | Fatty acid | 6-Octadecanoic acid | C_18_H_34_O_2_ | 282.46 | + |  | Zhang and Wang (2006) | |  |
| 107 | Fatty acid methyl ester | Methyl myristate | C_15_H_30_O_2_ | 242.40 | + |  | Zhang and Wang (2006) | |  |
| 108 | Fatty acid methyl ester | γ- Methyl Linolenate | C_19_H_32_O_2_ | 292.46 | + |  | Zhang and Wang (2006) | |  |
| 109 | Fatty acid methyl ester | Methyl palmitoleate | C_17_H_32_O_2_ | 268.435 | + |  | Zhang and Wang (2006) | |  |
| 110 | Fatty acid methyl ester | Methyl palmitate | C_17_H_34_O_2_ | 270.45 | + |  | Zhang and Wang (2006) | |  |
| 111 | Fatty acid methyl ester | Methyl linoleate | C_19_H_34_O_2_ | 294.47 | + |  | Zhang and Wang (2006) | |  |
| 112 | Fatty acid methyl ester | Methyl linolenate | C_19_H_32_O_2_ | 292.46 | + |  | Zhang and Wang (2006) | |  |
| 113 | Fatty acid methyl ester | Methyl oleate | C_19_H_36_O_2_ | 296.49 | + |  | Zhang and Wang (2006) | |  |
| 114 | Fatty acid methyl ester | Methyl stearate | C_19_H_38_O_2_ | 298.50 | + |  | Zhang and Wang (2006) | |  |
| 115 | Fatty acid methyl ester | Methyl arachidate | C_21_H_42_O_2_ | 326.56 | + |  | Zhang and Wang (2006) | |  |
| 116 | Fatty acid methyl ester | Methyl erucate | C_23_H_44_O_2_ | 352.59 | + |  | Zhang and Wang (2006) | |  |
| 117 | Fatty acid methyl ester | Methyl behenate | C_23_H_46_O_2_ | 354.61 | + |  | Zhang and Wang (2006) | |  |
| 118 | Fatty acid methyl ester | Methyl cis-15-tetracosenoate | C_25_H_48_O_2_ | 380.66 | + |  | Sun (2015) | |  |
| 119 | Fatty acid methyl ester | Methyl cis-9-hexadecenoate | C_17_H_32_O_2_ | 268.44 | + |  | Sun (2015) | |  |
| 120 | Fatty acid methyl ester | Methyl -9-octadecenoate | C_19_H_36_O_2_ | 296.49 | + |  | Sun (2015) | |  |
| 121 | Fatty acid methyl ester | Methyl -8-octadecenoate | C_19_H_36_O_2_ | 296.49 | + |  | Sun (2015) | |  |
| 122 | Fatty acid methyl ester | Methyl 9,12,15-octadecatrienoate | C_19_H_32_O_2_ | 292.46 | + |  | Sun (2015) | |  |
| 123 | Fatty acid methyl ester | Methyl cis-11-eicosenoate | C_21_H_40_O_2_ | 324.54 | + |  | Sun (2015) | |  |
| 124 | Fatty acid methyl ester | Methyl trans-11-eicosenoate | C_21_H_40_O_2_ | 324.54 | + |  | Sun (2015) | |  |
| 125 | Fatty acid methyl ester | Methyl cis-11,14-eicosadienoate | C_21_H_38_O_2_ | 322.53 | + |  | Sun (2015) | |  |
| 126 | Fatty acid methyl ester | Methyl 11,14,17-eicosatrienoate | C_21_H_36_O_2_ | 320.51 | + |  | Sun (2015) | |  |
| 127 | Fatty acid methyl ester | Methyl 11,13-icosadienoate | C_21_H_38_O_2_ | 322.53 | + |  | Sun (2015) | |  |
| 128 | Fatty acid methyl ester | Methyl Cis-11-eicosenoate | C_21_H_40_O_2_ | 324.55 | + |  | Sun (2015) | |  |
| 129 | Fatty acid methyl ester | Methyl tetracosanoate | C_25_H_50_O_2_ | 382.67 | + |  | Sun (2015) | |  |
| 130 | Indole derivative | Sinalexin | C_10_H_8_N_2_OS | 204.25 | + |  | Yu (2005) | |  |
| 131 | Indole derivative | Brassilexin | C_9_H_6_N_2_S | 174.22 | + |  | Yu (2005) | |  |
| 132 | Indole derivative | 9-Methoxy-2- (methylthio) -4,9-dihydro- [1,3] thiazino [6,5-b] indole | C_12_H_12_N_2_OS_2_ | 264.36 | + |  | Yu (2005) | |  |
| 133 | Indole derivative | 9-Methoxy-2- (methylsulfiny-1) -4,9-dihydro- [1,3] thiazino [6,5-b] indole | C_12_H_12_N_2_O_2_S_2_ | 280.36 | + |  | Yu (2005) | |  |
| 134 | Indole derivative | 2- (Methylthio) -4,9-dihydro- [1,3] thiazino [6,5-b] indole | C_11_H_10_N_2_S_2_ | 234.34 | + |  | Yu (2005) | |  |
| 135 | Indole derivative | Methyl- (1H-indol-3-yl)- methylcarbamodithioate | C_11_H_12_N_2_S_2_ | 236.35 | + |  | Yu (2005) | |  |
| 136 | Indole derivative | Methyl- (1-methoxy-1H-indole-3-yl) - methylcarbamodithioate | C_12_H_14_N_2_OS_2_ | 266.38 | + |  | Yu (2005) | |  |
| 137 | Indole derivative | 1H-indoleglucosinolate | C_16_H_19_N_2_O_9_S_2_ | 447.05 | + |  | Yu (2005) | |  |
| 138 | Indole derivative | 1-Methoxy-1H-indoleglucosinolate | C_17_H_21_N_2_O_10_S_2_ | 477.48 | + |  | Yu (2005) | |  |
| 139 | Others | Vitamin B_1_ | C_12_H_17_N_4_OS^+^ | 265.35 | + |  | Yu (2005) | |  |
| 140 | Others | Vitamin B_2_ | C_17_H_20_N_4_O_6_ | 376.36 | + |  | Yu (2005) | |  |
| 141 | Others | Vitamin B_3_ | C_6_H_5_NO_2_ | 123.109 | + |  | Yu (2005) | |  |
| 142 | Others | Vitamin C | C_6_H_8_O_6_ | 176.12 | + |  | Yu (2005) | |  |
| 143 | Others | Daucosterol | C_35_H_60_O_6_ | 576.85 | + |  | Yu (2005) | |  |
| 144 | Others | β-Sitosterol | C_29_H_50_O | 414.71 | + |  | Yu (2005) | |  |
